# From Detection to Protection: Antibodies and Their Crucial Role in Diagnosing and Combatting SARS-CoV-2

**DOI:** 10.3390/vaccines12050459

**Published:** 2024-04-25

**Authors:** Anoop Kumar, Prajna Tripathi, Prashant Kumar, Ritu Shekhar, Rajiv Pathak

**Affiliations:** 1 Molecular Diagnostic Laboratory, National Institute of Biologicals, Noida 201309, India; 2 Department of Microbiology and Immunology, Weill Cornell Medical College, New York, NY 10021, USA; tripathiprajna@gmail.com; 3 R. Ken Coit College of Pharmacy, University of Arizona, Tucson, AZ 85721, USA; 4 Department of Molecular Genetics and Microbiology, College of Medicine, University of Florida, Gainesville, FL 32610, USA; 5 Department of Genetics, Albert Einstein College of Medicine, Bronx, New York, NY 10461, USA

**Keywords:** COVID-19, SARS-CoV-2, diagnostic, serological assays, SARS-CoV-2-specific antibodies, immunotherapy, neutralizing monoclonal antibodies, convalescent plasma, vaccine, variants of concern (VOC), Omicron subvariants, antibody-dependent enhancement, original antigenic sin, long COVID

## Abstract

Understanding the antibody response to SARS-CoV-2, the virus responsible for COVID-19, is crucial to comprehending disease progression and the significance of vaccine and therapeutic development. The emergence of highly contagious variants poses a significant challenge to humoral immunity, underscoring the necessity of grasping the intricacies of specific antibodies. This review emphasizes the pivotal role of antibodies in shaping immune responses and their implications for diagnosing, preventing, and treating SARS-CoV-2 infection. It delves into the kinetics and characteristics of the antibody response to SARS-CoV-2 and explores current antibody-based diagnostics, discussing their strengths, clinical utility, and limitations. Furthermore, we underscore the therapeutic potential of SARS-CoV-2-specific antibodies, discussing various antibody-based therapies such as monoclonal antibodies, polyclonal antibodies, anti-cytokines, convalescent plasma, and hyperimmunoglobulin-based therapies. Moreover, we offer insights into antibody responses to SARS-CoV-2 vaccines, emphasizing the significance of neutralizing antibodies in order to confer immunity to SARS-CoV-2, along with emerging variants of concern (VOCs) and circulating Omicron subvariants. We also highlight challenges in the field, such as the risks of antibody-dependent enhancement (ADE) for SARS-CoV-2 antibodies, and shed light on the challenges associated with the original antigenic sin (OAS) effect and long COVID. Overall, this review intends to provide valuable insights, which are crucial to advancing sensitive diagnostic tools, identifying efficient antibody-based therapeutics, and developing effective vaccines to combat the evolving threat of SARS-CoV-2 variants on a global scale.

## 1. Introduction

The Severe Acute Respiratory Syndrome Coronavirus 2, commonly known as SARS-CoV-2, is a novel coronavirus that emerged in late 2019, causing the global COVID-19 pandemic, which had a profound impact on the entire human population worldwide [1]. The International Committee on Taxonomy of Viruses (ICTV) classified the Coronaviridae family into three subfamilies. One of these subfamilies, Orthocoronavirinae, encompasses four genera of viruses: *Alphacoronavirus*, *Betacoronavirus*, *Gammacoronavirus*, and *Deltacoronavirus* [2]. Among them, seven species have been identified as causing or being associated with respiratory infections in human beings, and also possess potential to infect other vertebrates. These species include *Alphacoronavirus* HCoV-229E and HCoV-NL63; *Betacoronavirus* HCoV-OC43 and HCoV-HKU1; Middle East Respiratory Syndrome Coronavirus (MERS-CoV); Severe Acute Respiratory Syndrome Coronavirus (SARS-CoV); and SARS-CoV-2 [3]. Out of these seven pathogenic coronaviruses (CoVs), three have caused outbreaks in human populations: SARS-CoV, which accounted for 774 deaths worldwide during 2002–2004 [4]; MERS-CoV, emerging in 2012 and leading to 935 deaths to date [5]; and SARS-CoV-2, which emerged at the end of 2019 and was declared a pandemic by the World Health Organization (WHO) from its outbreak until May 2023 [1]. While the four human-infecting CoVs (HCoV-229E, HCoV-HKU1, HCoV-NL63, and HCoV-OC43) typically result in mild symptoms, SARS-CoV, MERS-CoV, and SARS-CoV-2 exhibit a spectrum of clinical manifestations that range from mild to severe, including fatal outcomes. Disease severity is influenced by the specific viral variants involved, the extent of viral exposure (multiplicity of infection), and the efficacy of a host’s innate and adaptive immune responses in terms of combating an infection. Mortality rates vary significantly among these three coronaviruses, with SARS-CoV having a mortality rate of 10–15%, MERS-CoV displaying a mortality of up to 37% or higher, and COVID-19 displaying a mortality rate ranging from 2% to 4%, although it surpasses 13% in some countries and varies significantly based on the viral genetic variants involved [6].

**Figure 1:**
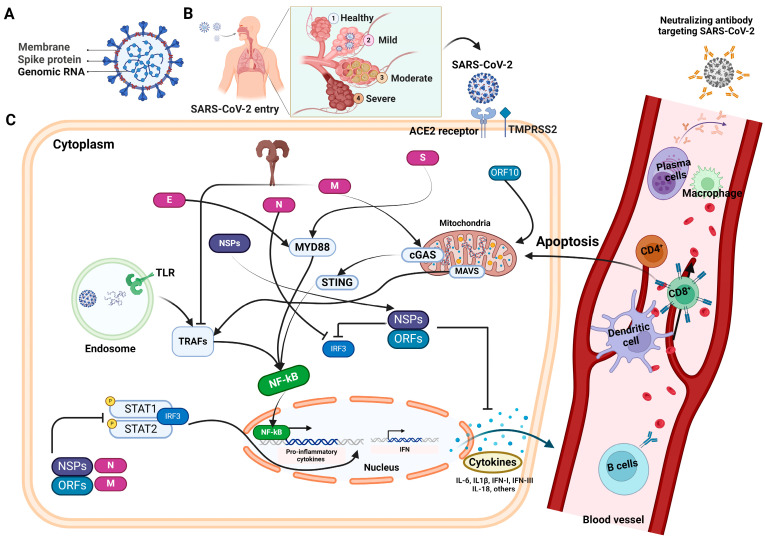
The interplay between SARS-CoV-2 infection and the host immune response: (**A**) structure of the SARS-CoV-2 virus; (**B**) entry of SARS-CoV-2 into the respiratory tract, leading to four different conditions: asymptomatic, mild infection, moderate infection, and severe infection; (**C**) interaction between SARS-CoV-2 proteins, such as open-reading frame (ORF), envelope (E) protein, membrane (M), spike (S) glycoprotein, and the nucleocapsid (N) protein, with host proteins regulating the immune response. The cytokine responses recruit dendritic cells and CD8^+^ killer T cells, initiating the adaptive immune response, which includes using CD8^+^ T cells, CD4^+^ T cells, and B cells to produce neutralizing antibodies (created with BioRender.com, accessed on 12 March 2024).

The SARS-CoV-2 is an enveloped virus, comprising a single-stranded positive-sense RNA genome, ranging in size from 29.7 kilobases (kb) to 29.9 kb. This encodes a 9860-amino acid-long polyprotein from the gene fragments, forming both structural and nonstructural viral proteins [7] (Figure 1A). In response to the challenges posed by COVID-19, significant efforts have been devoted to the development of diagnostics, treatments, and vaccines aimed at effectively managing the spread and impact of the virus. Currently, the most common diagnostic methods for SARS-CoV-2 infection are based on either the detection of virus-specific nucleotide sequences or the detection of virus-specific antigens and immunoglobulins [8]. In the current scenario, quantitative reverse transcription polymerase chain reaction (qRT-PCR) represents the gold standard for SARS-CoV-2 testing because of its high sensitivity and specificity. Despite being the current gold standard method for SARS-CoV-2 testing, qRT-PCR has several limitations. These include the need for ample viral RNA and optimal sample types, and the risk of false negatives due to missed replication time windows, especially during the early stages of recovery. Moreover, the proper collection, storage, packaging, and transportation of specimens are crucial for accurate results [9]. Due to all these reasons and the insufficient testing capacity of qRT-PCR, especially in low-resource countries, the necessity for rapid, accurate, and affordable diagnostic methods has been emphasized.

In a wide range of infectious diseases, specific antibodies play a pivotal role in viral infections, serving as both crucial defenders of the immune system and key markers in diagnosis due to their easy identification. Similar to other enveloped viruses, the exposed spike proteins of SARS-CoV-2 are the primary targets for recognition by host antibodies and the induction of adaptive immune response [10]. The retriggering of antigens by the B cells then stimulates the production of specific antibodies in order to neutralize the virus. In addition to their primary roles of defense and life-long surveillance, neutralizing antibodies (nAbs), which act against SARS-CoV-2, are also critical for the diagnosis, prevention, and treatment of COVID-19 [11]. Based on these crucial features of antibodies, serological assays that aim to detect antibodies against SARS-CoV-2 are being explored as potential alternatives or supplements to qRT-PCR, offering improved diagnosis for COVID-19 [12,13]. These assays and antibody tests capitalize on the detection of nAbs in the blood produced in response to specific viral antigens. The detection of antibodies has proven advantageous over qRT-PCR-based diagnosis due to the method’s reduced reliance on timely sampling and its capacity for detection even after the virus has cleared [14]. This is particularly useful for identifying individuals who may have had mild or asymptomatic infections, contributing to a more comprehensive understanding of the true prevalence of asymptomatic viral infection. Nonetheless, these tests exhibit drawbacks such as lower sensitivity compared to qRT-PCR and limited specificity due to possible cross-reactivity with other pathogens and autoantibodies in autoimmune disorders [15,16]. Furthermore, immunological methods may fail to detect antibodies during the early stages of infection, persisting up to 7 to 10 days post exposure, owing to their lower limit of detection. This characteristic renders them more efficient for use in identifying mid-to-late-stage infections compared to qRT-PCR [17,18]. Studies on the dynamics of the production of antibodies against SARS-CoV-2 have provided valuable insights into the natural course of infection, immune response timelines, and the effectiveness of antibodies in clearing the virus. The ability to assess the prevalence and persistence of antibodies in a population has also contributed immensely to epidemiological surveillance, the evaluation of vaccination campaigns, and, most importantly, the development of targeted public health strategies [19].

In addition to their role in diagnosis, antibodies are pivotal for the development of vaccines against SARS-CoV-2. Most COVID-19 vaccines are based on generating nAbs against viral antigens, specifically targeting the receptor-binding domain (RBD) of the spike (S) protein [20]. Multiple COVID-19 vaccines have now been developed, including mRNA vaccines produced by Moderna and Pfizer-BioNTech, as well as several adenovirus vector-based vaccines such as Sputnik-V and Oxford–AstraZeneca [21,22]. The mRNA vaccines developed by Pfizer-BioNTech and Moderna deliver mRNA into cells. This is translated by the host cells into a modified form of the virus’s spike protein, triggering an immune response [23]. However, in a study conducted by Fraiman et al., the association between the Pfizer-BioNTech COVID-19 vaccine BNT162b2 and the Moderna COVID-19 vaccine mRNA-1273, which both had serious adverse events in placebo-controlled, phase III randomized clinical trials (NCT04368728 and NCT04470427), was examined. The findings indicated that the Pfizer trial showed a 36% higher risk of serious adverse events among vaccinated individuals, while the Moderna trial showed a 6% higher risk [24]. The AstraZeneca and Johnson & Johnson vaccines employ replication-deficient adenoviruses as vectors in order to carry the genetic material for encoding the coronavirus’ distinctive spike protein. Following entry into the host cell, the recombinant adenovirus undergoes transcription and translation processes, thereby initiating the synthesis of viral antigenic proteins. Consequently, the immune system is stimulated to generate antibodies that target both adenovirus antigens and the SARS-CoV-2 spike protein, thus fortifying defenses against potential natural infections in the future [25,26]. The recombinant spike protein-based vaccine, NVX-CoV2373 (Novavax), is more straightforward and directly presents the S-protein subunit to the host by inducing the humoral immune response, leading to the induction of antibodies against SARS-CoV-2 [27]. The significance of all these vaccines lies in their ability to induce the production of nAbs, Th2-mediated humoral immune response, and memory cells, providing protection against severe illness and reducing transmission. Nevertheless, various concerns, such as vaccine efficacy, and potential side effects, remain unresolved due to different variants of the virus and the genetic diversity of the human population. Therefore, to further improve vaccine efficacy, safety, and immunogenicity, ongoing efforts are aimed at monitoring the levels and persistence of antibodies produced post vaccination.

Furthermore, monoclonal antibodies (mAbs), synthesized to mimic naturally occurring antibodies, have emerged as promising therapeutic tools for treating COVID-19-infected individuals, especially in cases with mild-to-moderate illness and poor prognosis [28,29]. Most mAbs target overlapping epitopes on the receptor-binding domain and motif (RBM) of the SARS-CoV-2 spike protein, effectively preventing viral entry by inhibiting S1 engagement with the ACE2 receptor. Additionally, some mAbs can trigger immune responses, such as antibody-dependent cell-mediated cytotoxicity and cellular phagocytosis, assisting in the removal of infected cells and providing adaptive immunity [30]. Overall, the significance of antibody-based treatments lies in their potential to reduce the severity of symptoms, prevent hospitalization, and shorten the duration of illness in certain individuals. However, the emergence of highly contagious variants continues to diminish the efficacy and utility of the available vaccines and antibody-based treatments against these new variants. Additionally, the phenomenon of antibody-dependent enhancement (ADE), defined by the possible post-infection or post-vaccination entry of immune complexes into cells with Fc receptors, as observed previously during pre-clinical studies of SARS-CoV and MERS-CoV vaccine development, has been a significant concern for the advancement of COVID-19 vaccines [31,32]. Since the onset of the pandemic, the approaches to diagnosing, treating, and vaccinating against COVID-19 have continually evolved, driven by the emergence of novel virus variants worldwide, and antibodies targeting SARS-CoV-2 have been instrumental in shaping these strategies. In conclusion, antibodies are indispensable tools for understanding, diagnosing, and combating SARS-CoV-2.

This review offers a comprehensive exploration of SARS-CoV-2-specific antibodies and their significance in diagnosing, preventing, and treating SARS-CoV-2 infection. Initially, we delve into the landscape of current antibody-based diagnostics and serological assays for SARS-CoV-2 detection, discussing their strengths, clinical utility, and limitations. Additionally, we emphasize the therapeutic potential of SARS-CoV-2-specific antibodies, highlighting their role as promising vaccine candidates and their protective efficacy. We evaluate recent advancements and assess the clinical efficacy of various immunotherapeutic strategies centered on SARS-CoV-2-specific antibodies. Moreover, we address the limitations and challenges within this domain, providing valuable insights that are crucial for developing highly sensitive diagnostic tools and identifying efficient vaccine candidates in order to effectively combat the emergence of new SARS-CoV-2 variants worldwide.

## 2. Antibody Response against SARS-CoV-2: Overview and Its Kinetics

Antibodies combat viral infections through several mechanisms, one of which involves inhibiting the interaction between the viral glycoproteins present in enveloped viruses and the specific receptors on host cells, thereby impeding viral entry into the target host cells [33] (Figure 1). Coronaviruses possess four major structural proteins: the spike (S) glycoprotein, envelope (E), membrane (M), and nucleocapsid (N) protein. Meanwhile, the other open reading frames (ORFs) of SARS-CoV-2 are found to encode for nonstructural proteins [34,35]. The infectious entry of SARS-CoV-2 into host cells is facilitated by the interaction between the viral S protein and the ACE2 receptor on the cell surface. ACE2 is expressed in endothelial, gastrointestinal, and respiratory cells. Type II transmembrane serine protease TMPRSS2 serves as the main host protease, facilitating S protein activation on primary target cells and initial viral entry [36,37]. Coronaviruses utilize a vast array of RNA-synthesizing and RNA-processing enzymes for genome expression and replication. The N protein of SARS-CoV-2, which binds to viral RNA, also plays a central role in RNA transcription and replication, and potentially affects the cell cycle processes of host cells. Similar to other viral infections, antibodies specific to the surface proteins of virus play a critical role in identifying, eliminating, and providing immunity against SARS-CoV-2. While the primary immune response to SARS-CoV-2 targets the N protein, protective immunity primarily relies on nAbs against the S protein. nAbs typically emerge against SARS-CoV-2 within 2–3 weeks post infection, with titers gradually decreasing in most recovered individuals after 5–8 weeks, but remaining detectable for up to eight months, which is contingent upon the initial virus titers [38].

Multiple studies have investigated antibody response kinetics in COVID-19 patients, revealing that most patients develop immune responses involving IgA, IgM, and IgG antibodies, targeting both S and N proteins of SARS-CoV-2 [12]. However, the N protein is highly conserved across all coronaviruses. Consequently, cross-reactions between pandemic and seasonal coronaviruses are quite possible [39]. Notably, both severe and nonsevere patients exhibit heightened levels of total antibodies and specific IgA, IgM, and IgG responses [40,41,42]. Clinical data regarding COVID-19 antibody responses remain consistent, regardless of the SARS-CoV-2 antigens used in immunoassays, including those containing RBD, S1 subunits, full-length S, or N proteins [40,41,43]. However, certain investigations into the humoral response suggest that specific features may correlate with more severe disease outcomes or even mortality. Studies on the kinetics of antibody responses commonly indicate that both IgG and IgM antibodies increase within the initial three weeks post symptom onset (PSO), with nearly all patients showing seroconversion and peak antibody levels by the third and fourth weeks [44]. Following this peak, IgM antibody levels begin to decline after the third week, while IgG levels remain stable for an extended duration of up to six or seven months [45]. Several studies have revealed that the median time for the seropositive conversion of specific IgG antibodies in COVID-19-confirmed cases is typically between 12 and 14 days after symptom onset. Notably, this timing aligns closely with the production of specific IgM antibodies, indicating a simultaneous production of both antibody types [46]. Another study analyzing 130 blood samples taken from 38 COVID-19 patients examined the kinetics of N- and S-specific IgM and IgG responses post symptom onset. Results showed that in non-ICU patients, seropositive rates of N-IgM, N-IgG, S-IgM, and S-IgG increased gradually within 1–3 weeks of onset. Peaks for N-IgM and S-IgM were observed in the second week, while N-IgG and S-IgG continued rising into the third week, reaching 100% seropositivity. However, some patients showed declining rates for N-IgM and S-IgM, likely due to the switch from IgM to IgG, potentially aiding in the generation of more effective antibodies against virus infection [44].

While some studies [44,45] did not find any significant differences among the three types of patients (asymptomatic, pre-symptomatic, and symptomatic) in this regard, two additional analytical studies highlighted the significant percentage of early responders among hospitalized [42,47] and severe patients [48]. Although nAb titers are found to fluctuate for as long as six months, a considerable decline is observed over time, according to research by Kim et al. [49]. The results of the study were found to be consistent, even after the segregation of the cohorts by age, disease severity, and sex, with no disparities detected in the reduction of nAbs over time in the groups compared. An unusual finding was the asymptomatic group’s relatively low but persistent nAb titer over time, in contrast to the other disease severity groups. According to the study, the vaccination of individuals who have recovered from COVID-19 increases the body’s natural antibody titer, which may further decrease the likelihood of reinfection. Moreover, predicting both natural and vaccine-induced immunity against reinfection requires the determination of the sequential nAb titers present in recovered individuals [49].

## 3. Profiling of Neutralizing Humoral Responses

The four essential structural proteins in SARS-CoV-2, namely, S, N, M, and E proteins, act as the potential targets of the antibody response, with, S and N proteins considered to be the primary targets of humoral response [46,50] (Figure 1C). The E and M proteins are extremely important in the process of virus assembly [51], and it has been proven that the N protein is essential for viral RNA production [52]. Conversely, the S protein is an essential component in the process of SARS-CoV-2 attachment and penetration into the host cells [53]. Humoral responses to various viral proteins are quickly elicited and exist in most infected individuals. The extent of these responses varies greatly between individuals and is positively correlated with the severity of COVID-19 infection [54]. The selection of the antigen is of the utmost importance in terms of harnessing virus-specific antibodies to detect SARS-CoV-2 infection and advance therapy and vaccination alternatives. In developing antibody tests, the critical factor for achieving high sensitivity and specificity lies in the careful selection of diagnostic antigens [55]. Moreover, directing attention toward the proteins that are essential for viral entry could potentially exert a substantial influence on the advancement of therapeutic interventions or vaccination strategies.

Many studies have discovered interesting SARS-CoV-2 antibody sequences while isolating and characterizing B-cell clones that express nAbs from recovered infected individuals. With few exceptions, the results concur, showing that a limited number of B clones drive the humoral immune response [56,57,58] and that patients often share heavy (VH) and light (VL) variable gene classes [58,59]. These public clonotypes are associated with distinct antibody classes based on the portion of S-protein detected [60]. The somatic hypermutations and complementarity determining region 3 (CDR3) lengths vary, even though SARS-CoV-2 infections have longer CDR3 sequences [57,61]. Kuri-Cervantes et al. examined these traits in connection with disease severity [61]. During acute COVID-19, plasmablasts expanded significantly, a phenomenon which is strongly associated with an oligoclonal growth of antibody clones in the B-cell repertoire, indicating that many of these large clonal expansions occur in the plasmablast pool. Mild patients, with one exception, exhibited less clone proliferation and more diversified repertoires. The antibody sequences of the largest B-cell clones in severe COVID-19 patients exhibited lengthy CDR3 regions, but showed remarkable variation in somatic hypermutation levels. Understanding the timing and context of B-cell activation and antibody responses is crucial, as highlighted by numerous studies on humoral immunity to SARS-CoV-2.

## 4. Immune Response following SARS-CoV-2 Infection and Vaccinations

The precise regulation of immune responses is essential for effectively eliminating pathogens, while any dysregulation of immunity can result in tissue damage and the onset of disease. The pathophysiology of COVID-19 is not only influenced by the viral load, but also by the extent of dysregulation in both innate and adaptive immune responses. In severe cases, discordant immune reactions contribute to delayed viral clearance, excessive inflammation, and tissue injury. These effects are not confined to the lungs, but can extend systemically, culminating in multi-organ failure. This disarray primarily stems from SARS-CoV-2’s ability to evade the innate antiviral response, which is mediated by type-I interferons (IFN-I), and persistent lymphopenia [62,63]. Recent research has compared the early immune and inflammatory effects of SARS-CoV-2 infection with those of other coronaviruses. In laboratory settings, both SARS-CoV and SARS-CoV-2 infected type-I and type-II pneumocytes, as well as alveolar macrophages. However, SARS-CoV-2 showed more efficient replication in pulmonary tissues [64]. Furthermore, in vitro studies revealed that SARS-CoV-2 increased the production of inflammatory chemokines such as interleukin-1 beta (IL-1β), IL-6, TNF-α, and interleukin-1 receptor antagonist (IL-1RA), while reducing IFN-I (α and β), and IFN-III responses. Correspondingly, COVID-19 patients exhibited elevated serum levels of these molecules, suggesting that SARS-CoV-2 possesses unique characteristics, including abilities to replicate in pulmonary tissue, evade IFN-I and IFN-III, trigger innate immune responses, and recruit adaptive immunity cells through cytokine activation. Thus, uncontrolled viral accumulation leads to aberrant activation and the recruitment of myeloid cells, triggering an exaggerated pro-inflammatory reaction that exacerbates immune-related damage [65]. Notably, individuals with severe COVID-19 exhibit heightened levels of circulating inflammatory cytokines, which is correlated with acute lung injury [66]. Given the intricate interplay between innate and adaptive immunity, impaired innate responses also impair adaptive immunity. Both the cell-mediated and humoral arms of adaptive immunity appear to be dysregulated following SARS-CoV-2 infection, resulting in the compromised activation of helper, effector, and cytotoxic T cells, as well as the dysregulation of immunological memory and antibody production.

All the COVID-19 vaccines approved so far demonstrate high efficacy in preventing severe disease upon infection. These vaccines efficiently engage with the innate immune system in order to initiate a robust adaptive immune response and the subsequent generation of immunological memory. However, vaccine breakthrough infections with new variants, coupled with the ongoing evolution of the virus, contribute to the continuation of COVID-19, despite efforts to end the pandemic. Nevertheless, notable differences exist among various vaccine types in the context of their immunogenic profiles.

(I) COVID-19 mRNA vaccines utilize mRNA to prompt the host’s cellular machinery in order to generate the SARS-CoV-2 S protein. Notably, this mRNA serves a dual purpose: instructing antigen production and triggering adjuvant activity by engaging with the innate immune system. However, modifications to the vaccine’s mRNA, including cytosine, adenine, and uridine methylations, are implemented to dampen recognition by immune receptors like toll-like receptors (TLRs) and RIG-I-like receptors (RLRs) [67]. This modification aims to prevent an exaggerated innate immune response, which could lead to vaccination side effects and impede the translation of the S protein, thereby potentially compromising the adaptive immune response to the antigen [68].

(II) The innate immune reactions activated by adenovirus vector (AdV)-based vaccines diverge from those prompted by mRNA vaccines due to the detection of DNA by distinct pattern recognition receptors (PRRs). Specifically, AdVs unveil pathogen-associated molecular patterns (PAMPs). These are recognized using TLR2 and TLR4, which are present on the cell’s surface, as well as by the presence of TLR9 within endosomes. Moreover, the detection of viral DNA can subsequently occur, leading to endosomal disruption through cytosolic DNA sensors like cGAS and via the inflammasome, triggering the release of IFN-I [69].

(III) NVX-CoV2373 stands out as being predominant among the authorized SARS-CoV-2 recombinant protein subunit-based vaccines. The formulation of this vaccine employs Matrix M, a saponin-based adjuvant, which swiftly stimulates innate immune cells, such as antigen-presenting cells. This occurs both locally at the injection site and in the draining lymph nodes (dLNs) [70]. Following this, the cytokines released by APCs attract more innate immune cells, initiating a cascade of localized immune responses. Additionally, Matrix M prompts the activation of the NLRP3 inflammasome, resulting in the release of IL-1β and IL-18, along with the generation and discharge of other pro-inflammatory cytokines [71].

(IV) Similar to the recombinant protein subunit-based vaccines, inactivated whole-virus vaccines also require adjuvants to enhance their effectiveness and immunogenicity. For instance, Covaxin incorporates a TLR7/8 agonist as its adjuvant. These agonists not only enhance the Th1 immune response but also dampen the Th2 response, which is advantageous for COVID-19 vaccines. Moreover, TLR recognition within the innate cell population has been associated with the early production of IFN-I, facilitating viral clearance and the generation of pro-inflammatory cytokines [72]. Unlike the first three vaccine categories, where the S protein acts as the primary immunogen, inactivated vaccines trigger broader immune responses. This is due to the inclusion of additional immunogenic proteins like the M, N, and E proteins. Consequently, there is a reduced focus on generating T cells that are specific to the S protein, but instead, a wider polyclonal T-cell response emerges, targeting various viral epitopes beyond just the S protein [73].

While the innate immune system initiates the early response to a vaccine, successful immunization necessitates the activation of both humoral and cellular adaptive immune responses. This entails generating nAbs and fostering the development of memory B cells and T cells. Dendritic cells (DCs), recognized as professional antigen-presenting cells (APCs), play a crucial role in linking these immune pathways. They achieve this by presenting vaccine-associated antigens to naïve CD4^+^ and CD8^+^ T cells through MHC-II and MHC-I molecules, respectively. Specific CD4^+^ T cells that recognize antigens can differentiate into follicular T helper cells (Tfh). These Tfh cells are instrumental in guiding B cells towards becoming high-affinity antibody-producing plasma cells and memory B cells. Through this mechanism, specific nAbs are generated against the virus, establishing immune memory [74].

An additional aspect worth considering is the potential role of trained immunity in safeguarding against COVID-19. This concept pertains to an immunological phenomenon where the key features include elevated cytokine production, alterations in metabolic pathways (such as protein kinase B, mammalian targets of rapamycin, hypoxia-inducible factor-1α, and nucleotide-binding oligomerization domain-containing protein 2-receptor signaling pathways), and epigenetic reprogramming upon subsequent exposure [75]. Trained immunity, characterized by a sustained enhancement in innate immune cell activity, has garnered attention in the context of vaccines like BCG, MMR, and OPV amid the COVID-19 pandemic [76,77]. These vaccines show promise in boosting the responsiveness of B and T cells to anti-COVID-19 vaccines. However, SARS-CoV-2 infection can instigate overly potent trained immunity responses. Animal studies suggest that BCG, administered intravenously, may offer protection against SARS-CoV-2 infection. Nonetheless, vaccines known for inducing trained immunity, such as BCG, MMR, and Shingrix, do not fully shield against SARS-CoV-2 infections. While they may lower the severity and mortality of COVID-19, the search for conclusive evidence necessitates large-scale randomized trials. BCG vaccination enhances the responsiveness of B and T cells to both mRNA- and adenovirus-based anti-COVID-19 vaccines. New COVID-19 vaccines trigger lasting trained immunity responses, yet SARS-CoV-2 infection can prompt excessive induction, leading to prolonged inflammatory complications [75].

## 5. Antibodies as Diagnostic Tools for SARS-CoV-2

Serological tests that target anti-SARS-CoV-2 antibodies are being recognized as promising alternatives, capable of complementing or substituting qRT-PCR-based diagnosis. Serological testing plays a crucial role in understanding the circulation of viruses and assessing infection containment strategies. In detecting SARS-CoV-2, the focus lies on its S and N proteins, with the latter being abundant during infection and highly immunogenic. However, using the N protein may lead to cross-reactivity with other coronaviruses, potentially impacting test specificity [39]. Undoubtedly, the S protein is an essential target for the activation of nAbs, which are generally regarded as the principal protective agents against SARS-CoV-2 infection. While serological testing plays a pivotal role in assessing immunity against reinfection and in guiding public health policies and workforce reintegration, careful consideration of antigen selection is vital for achieving accurate results when distinguishing SARS-CoV-2 antibodies. Antibodies that are specific to SARS-CoV-2 antigens constitute the primary focus of COVID-19 serological testing. IgG antibodies exhibit higher specificity towards antigens that emerge later, whereas IgM antibodies are the first to emerge in response to initial exposure. Numerous laboratories and companies have developed COVID-19 serological tests for IgG and IgM, serving various purposes. These tests can either validate results obtained from nucleic acid tests (NATs) or identify infected individuals who tested negative on NATs. Moreover, they are characterized by being low-cost, rapid, and suitable for broad screening at points of care (POC). In comparison to respiratory specimens, blood and serum samples exhibit less variability, enhancing the reliability of test results. Additionally, healthcare workers are at lower risk of contracting the virus from blood and serum samples than from patients, who are more likely to spread it through respiratory samples. Thus, these serological tests play a crucial role in comprehensive strategies for COVID-19 detection and management. Additionally, COVID-19 serological tests aid in gauging herd immunity and evaluating the efficacy of various vaccine candidates. However, they have limited specificity and sensitivity compared to qRT-PCR [78,79].

Serological testing is instrumental in comprehending the SARS-CoV-2 pathogen, aiding epidemiological research by evaluating virus control measures’ efficacy and understanding COVID-19’s viral circulation patterns. Serological techniques such as enzyme-linked immunosorbent assay (ELISA), chemiluminescent immunoassay (CLIA), and rapid diagnostic tests (RDTs) are utilized to specifically detect SARS-CoV-2 antibodies, particularly IgM and IgG [80,81,82] (Table 1).

### 5.1. Enzyme-Linked Immunosorbent Assay (ELISA)

ELISA is considered a benchmark for immunoassays. This is a versatile method used to detect and quantify various substances in specimens, including antibodies, antigens, proteins, glycoproteins, and hormones [83]. ELISA-based serology tests are employed to detect IgM and IgG antibodies that are effective against SARS-CoV-2, aiding in the determination of COVID-19-positive and -negative serum samples [84]. These tests have shown that specific IgM and IgG antibodies can begin to appear as early as the fourth day following the onset of symptoms [50]. Despite being a cost-effective and straightforward method, the efficiency of ELISA can be significantly enhanced by an automated workstation. However, it suffers from drawbacks such as time-consuming procedures, susceptibility to contamination, and the need for specialized infrastructure and skilled personnel [85,86]. Nonetheless, despite these challenges, ELISA remains indispensable in research, diagnostics, and diverse laboratory applications. Some of the ELISA tests developed for SARS-CoV-2 detection are discussed below:

#### 5.1.1. WANTAI SARS-CoV-2 Ab ELISA

The Wantai SARS-CoV-2 Ab ELISA diagnostic kit was developed by Beijing Wantai Biological Pharmacy Enterprise Co., Ltd., China, for the qualitative detection of total antibodies against the RBD of SARS-CoV-2, including IgM, IgG, and IgA. The Wantai SARS-CoV-2 Ab ELISA was designed to identify total antibodies, reflecting an immune response to SARS-CoV-2, in individuals suspected of prior infection, or to detect seroconversion in those with recent confirmed SARS-CoV-2 infection. The sensitivity of the Wantai SARS-CoV-2 Ab ELISA is found to be 79% in individuals showing symptoms for a minimum of 7 days [87]. Moreover, it has been shown that the sensitivity of the Wantai Ab escalates to 98–100% by the 14th day post symptom onset [88].

#### 5.1.2. EUROIMMUN Anti-SARS-CoV-2 ELISA Assay

The EUROIMMUN anti-SARS-CoV-2 ELISA assays, developed by EUROIMMUN Medizinische Labordiagnostika AG in Lübeck, Germany, are utilized for the semi-quantitative assessment of IgA and IgG antibodies, targeting the recombinant S1 domain of SARS-CoV-2. The Euroimmun anti-SARS-CoV-2 ELISA assay demonstrates strong sensitivity in detecting IgA and exceptional sensitivity in detecting IgG antibodies in samples collected at least 4 days post COVID-19 diagnosis by qRT-PCR. Similarly, it exhibits excellent specificity for IgG compared to IgA, with minimal cross-reaction. This was observed in only 2 out of 28 samples taken from patients infected with the common human coronaviruses NL63 and OC43 [89]. Various studies indicate low specificity, ranging from 72% to 90%, when considering both IgA and IgG components, but this improves significantly to 97.8% when focusing solely on IgG [90,91]. Given the subpar specificity for IgA, cautious interpretation is strongly advised, particularly when screening asymptomatic individuals [92].

### 5.2. Gold Immunochromatographic Assay (GICA)

The rapid and highly sensitive diagnosis of viral infections can be effectively achieved through the detection of serum-specific antibodies using the colloidal gold immunochromatography assay (GICA). GICA kits utilize colloidal gold-labeled N protein of SARS-CoV-2 to identify SARS-CoV-2-specific IgM and IgG antibodies present in the blood of individuals with COVID-19. This technique eliminates the need for specialized equipment and reagents, making it well suited for point-of-care testing. GICA for SARS-CoV-2 detects virus-specific IgM/IgG antibodies with a sensitivity of 71.1% and a specificity of 96.2%, making it a valuable and rapid diagnostic test for COVID-19 [93].

### 5.3. Chemiluminescence Immunoassay (CLIA)

In the chemiluminescence immunoassay (CLIA), recombinant antigen-coated magnetic beads are incubated with specific antibodies in a liquid sample to form immune complexes. Following this, an enzyme-labeled anti-antibody and substrate are introduced to initiate a chemiluminescence reaction, facilitating the detection of target analytes. The results are measured in relative light units (RLU) and can be used to identify SARS-CoV-2-specific IgA, IgM, IgG antibodies, and total antibodies within samples. The principle of CLIA resembles ELISA, but involves shorter incubation steps and eliminates the need for the use of a reagent to halt the enzymatic reaction, streamlining the process [94]. While the use of CLIA to target multiple antigens like N and S proteins demonstrates improved specificity compared to single-antigen assays, mitigating false positives remains a priority, highlighting the ongoing need to refine testing methodologies in order to enhance accuracy. The efficacy of CLIA use in detecting IgM was found to be less suitable, possibly due to its low concentration and rapid fluctuations in early- to mid-infection stages. Therefore, the measurement of total antibodies, rather than just IgM and IgG, is recommended for tracking improved infection progress and diagnosis [94]. Some luminescent assays developed for the diagnosis of SARS-CoV-2 are mentioned below:

#### 5.3.1. LIAISON SARS-CoV-2 S1/S2 IgG

The LIAISON SARS-CoV-2 S1/S2 IgG assay is a standardized automated chemiluminescent assay designed for the qualitative detection of IgG using the S1/S2 proteins native to SARS-CoV-2. This assay delivers results swiftly, providing accessibility within just 35 min; with an impressive throughput of 170 tests per hour, it ensures efficient testing processes. This assay has been examined in over 1500 samples, demonstrating sensitive and specific performance with a high-throughput capacity. Since the S1 and S2 proteins serve as targets for nAbs, the LIAISON assay is able to differentiate between neutralization (NT) assay-negative and NT assay-positive samples with good sensitivity and specificity [95]. However, in the study by Herroelen et al., significant lot-to-lot variation in the raw signals of two LIAISON kits was reported, suggesting the need to optimize cutoffs to achieve proper evaluation across different lots [96]. Additionally, Plebani et al. demonstrated the importance of redefining cutoffs for both LIAISON and iFlash assays to achieve the best negative likelihood ratio, emphasizing the necessity of considering variations and adjusting cutoffs for different assay lots to ensure result accuracy and reliability [97].

#### 5.3.2. Atellica IM SARS-CoV-2 Total (COV2T)

The COV2T assay is a chemiluminescent microparticle immunoassay (CMIA) that was developed to quantitatively detect total antibodies (IgG and IgM) against the RBD of SARS-CoV-2 in human serum and plasma, offering a fast and effective method for evaluating the presence of SARS-CoV-2 antibodies in biological samples. Results can be acquired from the assay within 10 min using the Atellica IM Analyzer, which is capable of processing up to 440 assays per hour [98].

#### 5.3.3. Abbott ARCHITECT SARS-CoV-2 IgG Immunoassay

The Abbott SARS-CoV-2 IgG assay is a fully automated indirect CMIA that was designed to detect IgG antibodies directed against the SARS-CoV-2 N antigen [99,100]. It has been observed that the sensitivity of the Abbott SARS-CoV-2 IgG assay depends on the timing of sample collection relative to the onset of symptoms. The sensitivity was found to be significantly higher for samples collected at ≥14 days post symptom onset compared to those taken earlier, with no significant differences observed between 14–20 days and ≥21 days. Therefore, sampling at 14 days post symptom onset was proposed as a suitable threshold for use retrospectively identifying COVID-19 patients [99].

#### 5.3.4. Yhlo Biotech iFlash 1800

The iFlash-SARS-CoV-2 is a CLIA-based assay that utilizes paramagnetic particles to detect IgM and IgG antibodies against the N and S proteins of SARS-CoV-2. However, its sensitivity was documented to be below 50% prior to day 8 after symptom onset, but it increased to 81.8% between days 9 and 10. Notably, sensitivity reached 100% after 15 days post symptom onset for IgG antibodies, indicating that performance improved with the time that elapsed following symptom onset [101]. This underscores the importance of considering sample collection timing in order to achieve accurate result interpretation.

#### 5.3.5. MAGLUMI 2019-nCoV IgM/IgG

The MAGLUMI 2019-nCoV IgM/IgG assays are also fully automated CLIA-based assays that are utilized to determine the presence of IgG and IgM antibodies against SARS-CoV-2 S and N proteins in human sera [102]. This automated system streamlines the process of antibody detection, offering efficiency and accuracy in assessing the immune response to SARS-CoV-2 infection.

### 5.4. Lateral Flow Immunoassay (LFIA)/Rapid Diagnostic Tests (RDTs)

Timely and accurate diagnostic testing for SARS-CoV-2 is crucial to achieving an effective COVID-19 response. In this regard, rapid point-of-care tests (also known as rapid diagnostic tests, RDTs) offer convenience and cost-effectiveness compared to traditional laboratory tests. These tests are used for qualitative antibody detection, do not require specialized operators or environments, and provide results quickly, allowing for the on-the-spot confirmation or ruling out of COVID-19 infection in both symptomatic and asymptomatic individuals [103]. RDTs that utilize lateral flow chromatographic immunoassays (LFIAs) are the most frequently used [81,82]. LFIA operates on the principle of liquid migration via a test membrane driven by capillary force, where immobilized capture antibodies interact with target analytes labeled for detection [104,105]. Various types of detection labels are presently employed, encompassing gold nanoparticles (GNPs), latex microparticles (LMPs), carbon nanoparticles (CNPs), magnetic nanoparticles (MNPs), quantum dots (QDs), silica nanoparticles (SiNPs), and europium nanoparticles (EuNPs) [104,106,107]. There is a wide array of commercially available LFIA tests, exhibiting diverse levels of sensitivity and specificity. These tests are specifically designed to detect antibodies specific to SARS-CoV-2. COVID-PRESTO and COVID-DUO are two commercially available SARS-CoV-2 rapid diagnostic tests for IgM-IgG. These were designed to detect IgG and IgM antibodies directed against the N-protein in whole-blood samples taken from the fingertip. In a study involving 238 qRT-PCR-positive COVID-19 patients, both RDTs showed 100% specificity, indicating that there were no false-positive outcomes. Furthermore, the sensitivity of these RDTs improved over time from symptom onset, with both achieving 100% sensitivity after 15 days [82]. Owen et al. evaluated 12 commercially available LFIA test kits, assessing their diagnostic accuracy and performance using 100 SARS-CoV-2-positive individuals and 105 pre-pandemic participants. The sensitivity of these tests against qRT-PCR varied, ranging from 30.3% to 74% for IgG, 21.2% to 67% for IgM, and 37.4% to 79% for IgM/IgG. In terms of specificity, the LFIA tests demonstrated ranges that ran from 82.9% to 100% for IgG, from 75.2% to 98% for IgM, and from 74.3% to 99.1% for IgM/IgG [108]. The varied assessments and recommendations highlight the importance of critically evaluating the test’s performance characteristics and considering specific use cases and populations when implementing rapid tests for COVID-19 diagnosis.

### 5.5. Neutralization Assay

The other classical assay used for assessing protective immunity against SARS-CoV-2 is the virus neutralization (NT) assay. Neutralization occurs when antibodies bind to viral particles, reducing their infectivity by preventing them from entering host cells. There are multiple serological tests available that assess virus–antibody interactions, but most of them predominantly detect binding antibodies rather than nAbs. However, a few assays, such as the plaque reduction neutralization test (PRNT), are considered the gold standard for measuring protective immunity as they assess virus neutralization during its attachment and entry into host cells [11]. The PRNT technique relies on the cytopathic effect (CPE), wherein the visual observation of damage to virus-infected target cells under a microscope indicates the effectiveness of antibodies. PRNT determines titers by measuring serum dilutions that result in reductions in plaque formation by 90% (PRNT_90_) and 50% (PRNT_50_) [109,110]. However, PRNT is time-consuming, requires skilled personnel, and can only be conducted in a BSL3 laboratory for handling live SARS-CoV-2 virus [111]. The live-virus-based microneutralization (MN) assay is also a highly sensitive and specific technique used to assess virus-specific nAbs in both human and animal sera [112]. Perera et al. conducted a study comparing the sensitivity and specificity of the MN assay and PRNT assay using 51 sera from 24 patients with confirmed COVID-19. Nevertheless, the sensitivity of the PRNT_90_ test was observed to surpass that of MN tests conducted with the standard 100-tissue-culture infectious-dose challenge [113]. However, neutralization assays using wild-type viruses may face limitations when testing plasma instead of serum from clotted whole blood. Plasma frequently contains heparin as an anticoagulant, which can inhibit SARS-CoV-2 entry into cells, potentially leading to inaccurate results in assays utilizing heparin-containing plasma samples.

Pseudovirus-based neutralizing assays (PBNAs) and surrogate virus neutralization tests (sVNT) are two safer alternatives to traditional virus-based assays for detecting the presence of nAbs against viruses like SARS-CoV-2. PBNAs use viruses without virulent components, requiring only a BSL2 laboratory, while sVNT operates within 1–2 h in a BSL2 lab without live-virus involvement [114,115]. Validation studies of sVNT have shown high sensitivity (95–100%) and specificity (100%) [115]. However, the PBNA method is restricted to evaluating the neutralization activity of serum- or monoclonal antibody-based treatments. Unlike wild-type virus assays, which can evaluate various antiviral compounds, PBNA’s scope is narrower because the pseudovirus lacks the CoV’s replicative machinery, which is targeted by these compounds. Given these considerations, there is an immediate demand for a high-throughput assay to quantify SARS-CoV-2 nAbs. Such an assay would greatly aid in COVID-19 serodiagnosis, convalescent plasma therapy (CPT), and the development of vaccines.

### 5.6. Biosensor-Based Technologies

Biosensors are analytical devices that integrate biological recognition molecules, such as enzymes, antibodies, or nucleic acids, with transducers in order to detect and provide the digital outputs of interacting analytes. Emerging biosensor technologies, like CRISPR-Cas9-based, nucleic acid-based, aptamer-based, nanoparticle-based electrochemical, electrochemiluminescence (ECL)-based, and plasmonic-based biosensors, hold considerable promise for the rapid detection of SARS-CoV-2. These biosensors offer cost-effective, sensitive, rapid, miniaturized, and portable platforms compared to conventional laboratory-based methods, presenting promising alternatives to traditional diagnostic assays [116,117]. Field-effect transistor (FET)-based biosensors represent a cutting-edge technology in biosensing, involving the application of graphene sheets, which are coated with a targeted antibody against the spike protein of SARS-CoV-2. The FET biosensor device showcased remarkable sensitivity. It was capable of detecting concentrations of the SARS-CoV-2 spike protein at exceedingly low levels, detecting 1 fg/mL in phosphate-buffered saline and 100 fg/mL in the clinical transport medium [118]. These innovative biosensor technologies contribute to the ongoing efforts in the rapid testing of mass populations to assess for COVID-19, providing potential solutions for the sensitive and efficient detection of the virus. However, there are some additional non-labeling techniques, such as SPR, Quartz-Crystal Microbalance (QCM), and Surface-Enhanced Raman Scattering (SERS), that have shown promising progress in biosensor research for viral samples, including RNA viruses like influenza, SARS-CoV-1, Ebola, MERS, Zika, and dengue [116,119,120]. These techniques enable the detection of host genetic biomarkers and specific nucleic acid sequences, offering the potential for the development of new biosensors for COVID-19 detection.

## 6. SARS-CoV-2 Variants of Concern (VOCs) and Their Impact on Diagnostics

Following the initial global dissemination of the wild-type SARS-CoV-2 strain, a multitude of mutant variants have arisen. The WHO classifies mutations with enhanced fitness as variants of interest (VOI), variants of concern (VOC), or variants under monitoring (VUM). Among these SARS-CoV-2 variants, VOCs pose an imminent threat due to their heightened transmissibility and disease severity. The current nomenclature used for easier recognition of VOCs includes Alpha (B.1.1.7), Beta (B1.351), Gamma (P.1), Delta (B.617.2), and Omicron (B.1.1.529) [121,122,123]. The Alpha, Beta, and Gamma variants, initially identified in the UK, South Africa, and Brazil, respectively, all carry the N501Y mutation. This mutation has been associated with a higher affinity of the spike protein for the human ACE2 receptor [124]. Beta and Gamma mutants carry the E484K mutation, which makes them more likely to evade blocking antibodies [125,126]. Additionally, Beta and Gamma variants exhibit changes in the K417 residue of the RBD, with Beta having a substitution of K417N and Gamma having a substitution of K417T. These variations have the potential to affect the binding of antibodies [124]. Furthermore, the Delta variant also shows distinct genetic variations (L452R, T478K, and P681R), which could enhance viral infectivity and fusion [127].

The Omicron variant, initially identified in Botswana and South Africa in November 2021, encompasses multiple lineages, and there has been a continuous increase in new lineages of the variant along with their global spread. Over a brief span, multiple Omicron subvariants, notably BA.1, BA.2, BA.3, BA.4, and BA.5, have promptly surfaced alongside the progenitor Omicron variants (B.1.1.529). These subvariants, characterized by genetic similarities to BA.1, possesses distinct mutations and are correlated with heightened transmissibility, contributing to the ongoing escalation of global cases [121,123]. In the year 2023, a multitude of Omicron subvariants, notably including recombinant XBB sub-lineages like XBB.1.9, XBB.1.5, and XBB.1.16, exhibited considerable prevalence. The emergence of the XBB lineages, characterized by their recombination of BA.2-derived subvariants (BJ.1 and BM.1.1.1), resulted in the displacement of numerous Omicron subvariants. This lineage is distinguished by heightened transmissibility rates and adept immune evasion properties [128,129,130]. Notably, XBB.1.16 stood out as the predominant strain until it was superseded by EG.5 within the United States. Among its derivatives, EG.5.1 garnered attention due to three significant alterations (Q52H, F456L, and F486P) observed in its S protein [131]. An additional Omicron subvariant, BA.2.86 (Pirola), was initially identified in Denmark and subsequently surfaced in Israel and Michigan (USA) in August 2023. Since its initial detection, it has been documented in several countries including Canada, England, France, Portugal, and South Africa [132]. BA.2.86 is presumed to be a descendant of BA.2 and is characterized by 33 mutations in the S protein and 14 in the RBD in comparison to the BA.2 variants [133]. The WHO designated BA.2.86 as a variant under monitoring (VUM) on August 17, 2023, due to the significant accumulation of mutations—among the highest recorded since the emergence of Omicron. Notably, aside from mutations shared with XBB.1.5 and BA.2.86, the RBD exhibits additional alterations, including I332V, K356T, V445H, N450D, N481K, A484K, and 483del, which are hypothesized to augment its capacity to evade immune responses. Furthermore, peculiar mutations within the N-terminal domain (NTD) may influence the antigenicity of BA.2.86. These findings suggest that BA.2.86 might possess increased transmissibility relative to existing XBB variants, such as EG.5.1 [122,133,134].

The COVID-19 pandemic has led to the development of qRT-PCR diagnostics, but these tests have limitations in terms of accurately detecting variations. Novel point-of-care diagnostic approaches have been developed to overcome these constraints. The WHO recommends using nucleic acid amplification tests such as qRT-PCR for diagnosis, while serological antibody testing and antigen-detecting rapid diagnostic tests (Ag-RDTs) provide rapid and cost-effective diagnosis. Antibody-based serological tests typically detect specific S- or N-directed antibodies in patient blood. However, mutations in viral proteins can affect test accuracy as patient antibodies may not recognize altered protein structures. Utilizing multiple fragments of key viral proteins like S and N to address this issue can enhance accuracy, especially against these emerging variants [135]. In such cases, monoclonal antibody-based assays targeting single epitopes may suffer from low sensitivity in rapidly evolving viruses, and so applying polyclonal antibodies to multiple epitopes could offer a more effective solution. Studies suggest that polyclonal anti-N antibodies may provide sensitivity against specific mutations such as H69/V70, N501Y, D614G, and D796H [136]. Jungnick et al. conducted a study investigating four different Rapid Antigen Test (RAT) kits, using cultured strains of various SARS-CoV-2 VOCs alongside non-VOC strains. The study found that these RAT kits effectively detected VOCs without cross-reactivity with recombinant N-proteins of other common human coronaviruses. However, it was noted that the N-mutations present in the B.1.1.529 (Omicron) variant might impact the accuracy of certain approved commercial antigen tests [135]. In another study, the sensitivity of the FDA-authorized OraSure InteliSwab™ COVID-19 rapid test was assessed against various SARS-CoV-2 variants, including Omicron. The test showed a consistent performance across different strains, with detection limits ranging from 3.77 × 10^5^ to 9.13 × 10^5^ RNA copies/ml. Notably, sensitivity remained unaffected when testing against the ancestral SARS-CoV-2 strain and all VOCs, including Omicron [137]. The consistent surveillance and evaluation of genetic variations’ influence on diagnostic tests, as well as cross-reactivity and the interference of other pathogens with the test performance, are crucial for understanding the disease’s spread and should be consistently investigated.

## 7. Antibodies as Therapeutic Agents against SARS-CoV-2: An Overview

Significant resources have been dedicated over the past year to the rapid development of diagnostic, preventative, and therapeutic approaches to COVID-19. Numerous researchers are diligently developing antibody-based tests for detecting SARS-CoV-2 and exploring the role of antibodies as therapeutic agents against SARS-CoV-2 [29]. Immunotherapeutic strategies, notably mAbs therapy and CPT, have garnered significant interest for their efficacy in bolstering patients’ immune responses and shielding them against viral infections [138]. mAbs have emerged as vital tools in the fight against COVID-19 due to their high specificity and adaptability. They play pivotal roles in combating the virus by halting disease progression and accelerating recovery, regardless of the patient’s immunity status. This development underscores the broader potential of therapeutic mAbs for use in treating various diseases, including SARS-CoV-2 infections. Here, we discuss various approaches that aim to fortify patients’ immune responses and shield them against viral infections, representing promising avenues for bolstering immunity and treatment efficacy.

### 7.1. Monoclonal Antibody-Based Therapies

Amidst the COVID-19 pandemic, numerous successful initiatives have been undertaken to investigate and formulate mAbs that target SARS-CoV-2. mAbs are laboratory-produced molecules that are engineered to serve as substitute antibodies. They hinder the interaction between the viral envelope and cell receptors, effectively preventing viral entry and replication, and safeguarding host cells from infection [139]. This mAb-based therapy is particularly valuable for individuals with weakened immune responses to vaccines, including people from the elderly and high-risk groups, and immunocompromised patients [140]. These antibodies, whether administered individually or as cocktails (combinations of two or more mAbs), have been widely endorsed by various authorities [141].

A multitude of randomized clinical trials were initiated to evaluate the effectiveness and safety of mAb therapy and prophylaxis. Initially targeting hospitalized patients, these trials were subsequently broadened to encompass outpatients, uncovering an overall moderate efficacy and favorable safety records across the spectrum. The effectiveness of mAbs has predominantly been observed in patients in early and mild disease stages, reflecting their antiviral role. However, their use faces challenges due to limited clinical efficacy and high costs, especially in low- to middle-income nations. The pandemic underscores the importance of combining diverse mAbs with non-overlapping mechanisms to counter immune evasion. The application of recombinant technology has also been instrumental in extending the half-life of these antibodies and minimizing their off-target effects [141]. The US Food and Drug Administration (FDA) and the European Medicines Agency (EMA) have authorized the emergency use of bamlanivimab and etesevimab, REGN-COV2 (casirivimab and imdevimab), and sotrovimab mAb for early treatment in at-risk outpatients and for post-exposure prophylaxis against SARS-CoV-2 (Table 2). However, restrictions were placed on the use of bamlanivimab and etesevimab, and REGN-COV2 (casirivimab and imdevimab), on 24 January 2022, and sotrovimab was withdrawn on 5 April 2022 [29,141].

Limited reviews and meta-analyses have been conducted to compare the efficacy of neutralizing mAbs before and after the emergence of different SARS-CoV-2 VOCs. However, most of these studies focus on only a subset of mAbs, rather than providing a comprehensive analysis of all the available options [142]. There is evidence suggesting that the clinical effectiveness of many neutralizing mAbs has decreased with the emergence of VOCs [143,144,145]. In an in vitro study by Yamasoba et al., the researchers investigated the susceptibility of new Omicron subvariants (BA.2.11, BA.2.12.1, and BA.4/5) to eight different mAbs (bamlanivimab, casirivimab, bebtelovimab, sotrovimab, cilgavimab, imdevimab, etesevimab, and tixagevimab). It was observed that the BA.2 spike protein with the R493Q mutation displayed only partial susceptibility to casirivimab and tixagevimab. Additionally, bamlanivimab, etesevimab, and imdevimab were found to be less effective against the new Omicron subvariants. Notably, bebtelovimab showed double the efficacy against BA.2 and all other Omicron subvariants compared to the parental Omicron variant (B.1.1) [144]. A recent study evaluated eight sub-variants of SARS-CoV-2, including XBB.1.16, XBB.1.5.24, XBB.1.9.1, XBB.1.9.3, CH.1.1, CL.1, BQ.1.1.45, and XBB.2.9, to test the effectiveness of various mAbs like Evusheld, Ronapreve, Bamlanivimab, Regdanvimab, Etesevimab, and Sotrovimab. While all mAbs showed strong neutralization against B.1.1.1., they largely failed against Omicron XBB, BQ, CL, and CH subvariants, except for Sotrovimab, which displayed reduced effectiveness against certain subvariants [146]. In another study, 72 immunocompromised patients were administered with Evusheld (tixagevimab/cilgavimab), and its effectiveness in neutralizing various SARS-CoV-2 subvariants (BQ.1, XBB.1.5, XBB.1.16, 20A.EU1, EG.5, and BA.5) was evaluated up to 30 days post treatment. The study revealed a significant reduction in Evusheld’s neutralizing activity against newer variants, like BQ.1, XBB.1.5, XBB.1.16, and EG.5 [147]. Qu et al. recently examined the effectiveness of mAb S309 in neutralizing various SARS-CoV-2 variants, including BA.2.86 and XBB-derived FLip, alongside BA.1, BA.2, BA.4/5, D614G, EG.5.1, and XBB.1.5. The results indicated that BA.2.86 exhibited lower immune evasion compared to FLip and other XBB variants. However, mAb S309 failed to neutralize BA.2.86, possibly due to a D339H mutation [134]. Recently, Liu et al. delved into the neutralization effectiveness of 28 potent spike-binding mAbs derived from individuals experiencing breakthrough infections with the Omicron sub-lineages BA.4 or BA.5. Their findings revealed a gradual decline in the neutralization capability of the BA.4/5 mAbs, ultimately resulting in a complete loss of function against recent XBB.1.5.70 variants carrying the notable ‘FLip’ mutations at positions 455 and 456 [148].

However, the available data on the safety and effectiveness of these treatments in vulnerable populations such as the elderly, high-risk individuals, and immunocompromised patients are limited, raising significant concerns regarding their suitability for these groups [149]. mAb therapies face significant limitations due to their high specificity and affinity, making them vulnerable to failure when even a minor mutation occurs in the targeted epitope. Given that existing mAbs primarily target regions prone to mutation in SARS-CoV-2, such as RBD and NTD, they are at risk of losing their effectiveness against emerging variants [150,151]. Therefore, developing mAbs that target conserved viral epitopes is crucial for establishing broad-spectrum antibody therapies.

### 7.2. Anti-Cytokine Antibodies

SARS-CoV-2 infection triggers an excessive immune response in the host, resulting in an exaggerated inflammatory reaction characterized by the abundant production of various cytokines and chemokines by the host cells [152,153]. While most COVID-19 patients experience mild or no symptoms, some individuals may experience severe manifestations of the disease [154] (Figure 1). In severe cases, these cytokine storms can result in various clinical complications, including acute respiratory distress, pulmonary edema, renal failure, acute liver injury, and multiorgan failure [155]. The activation of innate immunity and dendritic cells, accompanied by the excessive release of chemokines (e.g., CCL20, CXCL1, CXCL2) and cytokines (e.g., IL-6, TNF-α), is the initial consequence of SARS-CoV-2 invasion. This release subsequently stimulates the generation of B-cell and T-cell responses that are specific to SARS-CoV-2. Rapid viral clearance is attributed to T-cell responses against SARS-CoV-2, while serious or acute disease is correlated with a prolonged duration of the innate immune response [153,156]. Ineffective IFN I and III innate immunity, resulting in innate cell immunopathology and cytokine storm, are other crucial discoveries linked to severe/acute illness. GM-CSF, IL-7, IL-8, IL-9, IFN-γ, TNF-α, MCP1, MIP1A, MIP1B, G-CSF, and IL-6 are among the critical cytokines abundantly present during the SARS-CoV-2 cytokine storm, which is induced by macrophages and other innate immune cells [157,158,159]. In addition to coagulation and other severity-associated symptoms, the signature of these pro-inflammatory cytokines is readily apparent in patients with COVID-19 pneumonia [160]. Furthermore, it has been observed that inflammation can persist for several months following the elimination of the virus in a condition known as post-COVID-19 syndrome or long COVID, which manifests in numerous recovered patients [161]. Research indicates that administering dexamethasone, an anti-inflammatory corticosteroid, to hospitalized patients, whether on mechanical ventilation or not, significantly reduces mortality rates [162]. Several small-molecule drugs currently undergoing clinical trials offer potential benefits for COVID-19 patients alongside potential targets. However, streamlining the development of therapies aimed at these targets hinges on the identification of crucial cytokines.

### 7.3. Polyclonal Antibody Therapies

Polyclonal antibodies represent a heterogeneous collection of antibodies that recognize multiple epitopes on the viral envelope, demonstrating potent neutralizing effects during infection. Their ability to recognize and bind to multiple epitopes enables them to trigger a range of effector functions [163]. Polyclonal antibodies offer several advantages over mAbs, such as cost-effectiveness, rapid production, strong stability, high affinity, and lower sensitivity to antigen variations. However, their clinical utility is constrained by their tendency for cross-reactivity and lack of specificity compared to mAbs [164]. Despite these limitations, the findings on the effectiveness of using convalescent and recovered patients’ sera to neutralize COVID-19 in laboratory settings have led to the exploration of convalescent plasma and hyperimmune globulins as potential treatments for severe cases of the disease.

#### 7.3.1. Convalescent Plasma Therapy (CPT)

Convalescent plasma (CP), which is rich in antibodies harvested from recovered individuals, stands as the most accessible reservoir of SARS-CoV-2 nAbs. This source presents a viable option for passive immunotherapy in both infected patients and those susceptible to the virus [165]. The 1918 influenza pandemic underscored the effectiveness of CP use in combating infectious diseases. CP has also been proven successful in combating various emerging infectious diseases (EID), including H5N1, Ebola, SARS-CoV, and MERS-CoV outbreaks, demonstrating its therapeutic potential across different viral threats [166,167,168]. CP-containing nAbs offer a potential treatment avenue for COVID-19, providing passive immunity against the virus [169,170]. The FDA granted emergency use authorization for CP use in 2020 to treat severe or life-threatening cases of COVID-19. A study that was conducted across 7 medical centers in the USA and involved 103 COVID-19 patients examined the safety of convalescent plasma. The results showed that, within 28 days, 51.9% of patients in the convalescent plasma group experienced clinical improvement. Notably, among those with severe disease, 91.3% showed primary outcome improvement, while in the life-threatening category, 20.7% saw an improvement in primary outcomes. However, there was no significant difference observed in 28-day mortality or discharge time compared to the control group [171]. Another randomized clinical trial involving 475 patients was conducted between September 2020 and March 2022, and this compared the outcomes of administering convalescent plasma versus standard care. Among these patients, 237 received convalescent plasma, while 238 received standard care. Notably, 17.7% of the convalescent plasma group received a neutralizing antibody titer of 1:160, while 98.1% received glucocorticoids. By day 28, mortality was lower in the convalescent plasma group (35.4%) compared to the standard care group (45.0%), particularly among randomized patients within 48 h of initiating invasive mechanical ventilation [172]. Despite these promising results, the utilization of CP faces challenges such as storage issues and a limited supply of blood donors. Also, these approaches are not only expensive and time-intensive, but also fail to offer protection against inflammation. Moreover, the presence of non-nAbs raises concerns about ADE, exemplified by conditions like dengue hemorrhagic fever [173].

#### 7.3.2. Hyperimmune Globulins (HIGs)

Since the late 19th century, antibody-based therapies have evolved significantly. These encompass the use of pooled hyperimmune immunoglobulins (HIGs), concentrated from a pool of convalescent plasma from immunized donors, for intravenous (IVIG) or intramuscular/subcutaneous (IM/SCIG) administration [174]. HIGs would likely enhance safety and efficacy due to specific and accurately defined nAb titers. These IVIG nAbs boast higher titers, thus holding promise for widespread use in the treatment of numerous patients [175]. Nonetheless, the preparation and distribution of IVIGs require several months, prolonging the process of making them available to clinics and hospitals [176]. Since vaccinations usually take time to generate protective immunity, these HIGs could potentially complement vaccinations by offering temporary immunity while the body generates its own protective response [177].

Therefore, polyclonal immunoglobulin continues to be used in the treatment of various infectious diseases, including COVID-19. COVID-19 HIGs are found to exhibit strong binding to various key components of the SARS-CoV-2 virus, including the spike protein, RBD, NTD, and nucleocapsid proteins. Extensive testing using both pseudotype and authentic virus assays demonstrates broad-spectrum neutralization against multiple SARS-CoV-2 variants, such as alpha, beta, gamma, kappa, delta, and omicron, with a notable decrease in neutralization potency specifically observed against beta, delta, and omicron variants [178,179]. However, further clinical trials are needed to ascertain the efficacy and safety of passive COVID-19 vaccination against emerging VOCs.

## 8. SARS-CoV-2-Neutralizing Antibodies and Their Potential Role in Vaccine Development

### 8.1. Protective Roles of Neutralizing Antibodies against SARS-CoV-2 Infection

The neutralization of a virus can be briefly defined as the process of reducing its infectiousness by attaching antibodies to the surface of viral particles (virions) and thus preventing the viral reproduction cycle [11]. Generally, nAbs hinder the virus from attaching to cellular receptors; however, in certain instances, they may also impede the conformational changes necessary for the virus to fuse with the cell membrane or for proteolytic cleavage to occur [180]. Regarding enveloped viruses, the most recent phase that appears to be halted is membrane fusion, also known as entry into the cytoplasm. Conventionally, the function of nAbs is mediated by a region known as the fragment antigen-binding (Fab) area, while the effect of non-neutralizing antibodies is exerted close to the crystallizable area (Fc). The formation of nAbs, generated either by vaccinations or by previous infections, is an essential component in the process of providing protection against the SARS-CoV-2 virus [181]. The primary focus of nAbs for protective immunity lies on the SARS-CoV-2 S protein. The RBD, located within the S1 subunit of the trimeric S protein, enables viral attachment by specifically binding to the host cell receptor ACE2. This interaction between the RBD and ACE2 is pivotal for viral entry, emphasizing the significance of the S1 subunit of SARS-CoV-2 as a primary focus for vaccine development [182,183]. Efforts to assess vaccine efficacy (VE) and anticipate immune protection in individuals heavily relies on nAbs targeting the SARS-CoV-2 S protein [184]. Elevated levels of these antibodies have been linked to reduced risks of both SARS-CoV-2 infection and severe COVID-19 disease. Various studies have indicated that individuals with heightened titers of nAbs are less prone to experiencing symptomatic COVID-19 subsequent to either natural infection or vaccination [185,186,187,188]. Perry et al. have suggested in their systematic review that nAb titers could possibly serve as a correlate of protection (CoP) from SARS-CoV-2 [189]. The authors have also identified a strong association between antibody levels induced by vaccination and VE, even in the face of considerable diversity in vaccination schedules, serological testing methods, VE measurement criteria, and study populations. Furthermore, almost all existing vaccines, biologic therapies with mAbs, and convalescent plasma used to control SARS-CoV-2 infection are based on the utilization of nAbs, which can prevent infection by interfering with various stages of the viral replication cycle [181,190].

### 8.2. SARS-CoV-2 Vaccine Efficacies and Specific Immune Responses

**Figure 2:**
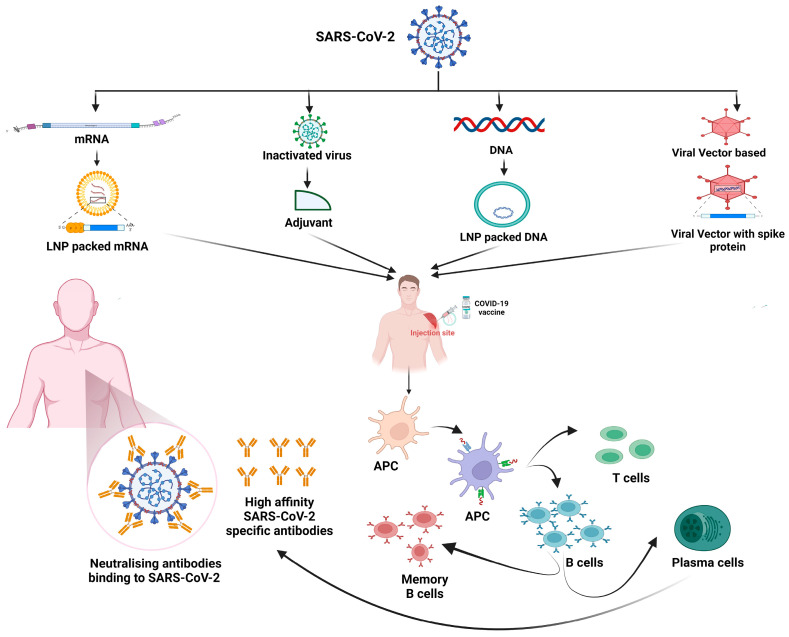
Illustration of different types of COVID-19 vaccine development and the generation of immune response (created with BioRender.com, accessed on 12 March 2024).

Neutralizing antibodies serve as crucial indicators of the immune protection provided by vaccines against symptomatic SARS-CoV-2 infection [186,187]. As the world has witnessed, various types of COVID-19 vaccines are highly effective in preventing SARS-CoV-2 infection and in reducing symptoms after infection. Almost all of these vaccinations provoke systemic immune responses; however, differences in the immune responses caused by different immunization regimens are readily evident [191] (Figure 2). Vaccine design is a crucial process, leading to an optimal immune response, and the selection of the antigen and platform, the need for adjuvants, the formulation, and the method of delivery are some of the determining factors [192]. The composition of COVID-19 vaccines consists of certain proteins, namely spikes S1 and S2, nucleocapsid protein, membrane protein, and envelope protein, which constitute the structure of the SARS-CoV-2 virus. However, most of the vaccines developed to fight against SARS-CoV-2 incorporate the S protein [193]. COVID-19 vaccines can be classified into four primary categories: attenuated whole-virus vaccines (Columbia, BBVBP-CorV, and Covaxin), protein-based vaccines (Nuvaxovid), viral vector vaccines (JCOVDEN, Vaxzevria, and Sputnik V), and nucleic acid vaccines (BNT162b2 and Spikevax) [194,195] (Figure 2).

The strategy of rolling out mass vaccination campaigns to distribute SARS-CoV-2 vaccines demonstrated remarkable effectiveness in terms of reducing the impact of the COVID-19 pandemic. Supplementing these immunization efforts with the surveillance of the immune response was crucial for achieving comprehensive infection management. Clinical studies have established that vaccines based on mRNA, adenoviral vectors, and proteins demonstrate efficacies ranging from 74% to 95% against symptomatic disease [196,197,198,199]. Furthermore, the IgA and IgG responses against S antigens were shown to be higher in infected individuals who had received vaccinations, compared to their peers who had not been vaccinated. It was demonstrated that this disparity in response continued to exist five months after vaccination. Research conducted by Bates and colleagues demonstrated that an increase in the humoral immune response takes place regardless of whether an infection is acquired before or after vaccination. This increase is also associated with the neutralization and binding of antibodies [200]. However, protection against infection diminishes over time due to decreasing immune response and the appearance of variants that can evade elimination by the antibody response. In a meta-analysis by Zaeck et al., the effectiveness of various vaccines diminished over time and they were found to be 86% effective in reducing death after 168–195 days, 79% effective in reducing hospitalizations after 224–251 days, and 47% effective in reducing infections after 280–307 days [201]. In the same study, the authors reported that booster doses enhance the effectiveness of all forms of SARS-CoV-2 vaccines, resulting in a 70% increase in protection against infection and an 89% increase in protection against hospitalization [201]. Therefore, determining whether booster doses are necessary requires an understanding of the duration of protection provided by COVID-19 vaccines.

Other studies led by the Oxford COVID vaccine trial group have estimated the VE of the ChAdOx1 nCoV-19 vaccine (University of Oxford/AstraZeneca), which is an adenoviral vaccine with a complete SARS-CoV-2 spike insert. Their findings illustrate that estimates of VE against symptomatic COVID-19 infection were higher in subgroups that displayed elevated pseudovirus neutralization antibody titers or increased levels of anti-spike IgG. These observations were made in clinical trials of ChAdOx1 nCoV-19 in adults [202]. Furthermore, elevated levels of all immune markers were inversely proportional to the risk of symptomatic infection. The overall VE against symptomatic infections was estimated to be about 80%, while for asymptomatic infections, it was reported to be 27% [203]. A similar study by Gilbert et al. reported the CoP associated with the Moderna phase 3 vaccine trial, and the findings appeared very similar to those of the above-mentioned studies. However, the total binding antibody titers and pseudovirus nAb titers were higher after vaccination with the Moderna vaccine [204]. Therefore, these findings indicate the strong inverse correlations between all assessed binding antibody (bAb) and nAb markers with COVID-19 risk and show their direct correlations with VE. The correlates of protection highlighted in these studies can be applied to infer efficacy from immunogenicity data for novel vaccines in cases where results on clinical efficacy are not yet available.

The efficacy of existing immunizations has been questioned due to several variables, including the emergence of mutations in the SARS-CoV-2 virus. Studies suggest that while there might be a small decrease in effectiveness against certain variants, immunizations, particularly mRNA vaccines, remain advantageous in terms of preventing serious illnesses caused by mutations [201,205]. However, continued research and surveillance are crucial for optimizing the efficacy of immunizations and adapting them to new and emerging variants.

### 8.3. Antibodies as Predictive Biomarkers for Vaccine Efficacy

The screening of the immune system over an extended period has revealed that responses to pathogens and vaccines are extraordinarily multifactorial and involve a multitude of diverse factors. The presence of vaccine-induced nAbs has shown a strong correlation with preventing COVID-19 across various vaccine trials and meta-analyses [186,204,206,207]. Recommendations from both the FDA and the International Coalition of Medicines Regulatory Authorities advocate for the use of vaccine-induced nAb titers as a measure of protection. This recommendation guides the authorization process for new variant vaccines and booster doses, drawing from immunogenicity studies conducted on historical clinical trials that were focused on clinical endpoints related to ancestral or early variants of concern. In a recent study [208], researchers evaluated the use of nAb titers such as CoP against COVID-19 in the mRNA-1273 vaccine trial (ClinicalTrials.gov # NCT04470427), as well as in the casirivimab + imdevimab mAb prevention trial (ClinicalTrials.gov # NCT4452318). While both the mRNA-1273 vaccine and casirivimab + imdevimab mAbs showed notable effectiveness in terms of preventing COVID-19 throughout the duration of the study, the proportionate influence of antibodies at the time of exposure varied. When nAb titers exceed 1000 IU50/mL, both the mRNA-1273 vaccine and casirivimab + imdevimab demonstrated a clinical efficacy of over 90% in preventing COVID-19. At this threshold, existing antibodies accounted for approximately 72% of the overall vaccine effect. Conversely, at lower titers (e.g., <100 IU50/mL), mRNA-1273 maintained its efficacy, while the effectiveness of the mAb combination, casirivimab + imdevimab, diminished. Various studies have supported the idea that the efficacy of vaccines is likely influenced predominantly by nAbs and memory antibody responses, while also being reinforced by T-cell responses [206,209,210,211]. These combined immune responses play a crucial role in safeguarding against severe cases of COVID-19, particularly in scenarios where SARS-CoV-2 variants manage to evade neutralizing antibody responses. Overall, the findings from the research of Follmann et al. endorse existing antibodies as a mechanistic indicator of protection in humans, with their significance varying depending on the level of antibody titer at exposure [208].

### 8.4. SARS-CoV-2 Variants of Concern (VOCs) and Their Impact on Vaccine Efficacy

The symptoms and severity of COVID-19 variants can differ due to variant-specific mutations and amino acid alterations, affecting their virulence and tissue tropism. However, identifying these mutations becomes challenging as variants evolve over time and is difficult in diverse populations with varying levels of immunity [212]. Predominantly, these variants fall within the SARS-CoV-2 lineage B, notably lineage B.1. Among them, the D614G variant stands out as one of the most widespread, harboring a mutation in the C-terminal region of the Spike 1 (S1) domain, positioned beyond the RBD. Despite some reports suggesting increased pathogenicity, sera from convalescent patients and individuals vaccinated with mRNA vaccines show comparable levels of neutralization of the D614G mutant compared to the ancestral strain, specifically with regard to lineage B or the wild-type strain [179,213,214]. Ongoing global vaccination efforts face challenges due to the presence of circulating SARS-CoV-2 VOCs. These variants possess distinct mutations in the RBD and have the potential to evade the neutralizing effects of antibodies [213].

Most COVID-19 vaccines were developed to treat the initial strains of the pandemic. However, the appearance of new VOCs, including Alpha, Beta, Gamma, Delta, and Omicron, posed a challenge to the efficacy of these vaccines (Table 3). Several studies have consistently found that VOCs have higher infectivity and a greater ability to evade the immune system compared to the original strains. Additionally, the effectiveness of vaccines against these VOCs is weaker and diminishes rapidly over time. It is crucial to determine whether immunization can provide protection against these variants, given their increased infectivity and mortality rates [124]. The increased dissemination of several SARS-CoV-2 VOCs underscores the critical importance of maintaining the surveillance of vaccine efficacy against the constantly evolving virus targets. The enhanced ACE2 affinity for the SARS-CoV-2 RBD is likely responsible for the heightened transmission rates of the Alpha lineage [215]. This is not attributable to the evasion of pre-existing antibodies in convalescent or vaccinated individuals [126]. Research indicates that variants carrying the E484K substitution substantially elevate the likelihood of immune evasion in both vaccinated individuals and those who have previously recovered from COVID-19. Undoubtedly, the incidence of illnesses linked to the Gamma variant, which includes the E484K substitution among other mutations, is escalating rapidly in Manaus, Brazil. This situation is persisting despite the population seropositivity rate having been 76% since March, 2020 [216]. Early vaccine trial data for Novavax against the Beta lineage in South Africa, which also encodes the E484K substitution, indicated a substantial decline in effectiveness within the realm of vaccination. The increased prevalence of new infections and the improved transmission characteristics attributed to the Delta variant provide further evidence of the critical nature of SARS-CoV-2 therapeutics [217].

Based on the current understanding of this issue, several randomized controlled trials (RCT) were conducted to assess VE against different clinical outcomes produced by the VOCs [218,219]. A meta-analysis study found that full vaccination offers robust protection against various clinical outcomes, with VE ranging from 86.8% to 96.0% against the Alpha variant [220]. Additionally, it provides moderate protection against infections caused by the Beta, Gamma, and Delta variants, with VE ranging from 70.9% to 72.8%. Furthermore, it offers strong protection against severe disease caused by the Delta variant, with VE ranging from 84.9% to 90.3%. However, the study showed limited protection against infection overall, with a VE of 23.5% (95% CI, 17.0–29.5), and moderate protection against severe disease caused by the Omicron variant, with VE ranging from 56.5% to 82.4%. Booster immunization can significantly enhance the level of defense against the Delta and Omicron variants, although not to the same extent as it does against the Delta variant. The meta-regression study revealed a decrease in VE against the Omicron variant over time. Additionally, the value of VE against hospitalization exhibited a relatively gradual fall compared to that of VE against infection [220].

In particular, the nAbs used, which were generated by vaccinations that were initially licensed, were less effective against newly emerging VOCs, which ultimately led to the adaptation of mRNA vaccines [221,222]. When original or adapted vaccine boosters are delivered at suitable intervals, there is clear evidence of vaccine-mediated protection, at least against severe COVID-19 [223,224]. In light of the same observation, Trombetta et al. conducted tests, using samples collected from both COVID-19 patients and vaccinated individuals, to gauge the impact and susceptibility of various serum samples to the Omicron variant BA.1 [225]. These samples were analyzed for their ability to bind to and neutralize both the original SARS-CoV-2 virus and the Omicron variant BA.1. In COVID-19 patients, there was a noticeable decline in Omicron-specific antibody response compared to their response to the wild-type virus. However, antibodies generated through either a triple homologous/heterologous vaccination regimen, or as a result of natural SARS-CoV-2 infection followed by a two-dose vaccine course, demonstrated the highest neutralization capacity against the Omicron variant BA.1. Collectively, the findings of the multiple studies mentioned above endorse the current approach of administering mRNA vaccines and boosters in order to augment antibody-mediated cross-protection and guard against emerging Omicron variants.

The latest iterations of the Omicron variant, BQ.1.1 (a subvariant of BA.5) and XBB.1.5 (a subvariant of BA.2), have developed mutations that enhance their ability to evade the immune system while still maintaining their affinity for receptor binding [226]. In Finland, to comprehend the dynamics of vaccine-induced nAbs following infection with various SARS-CoV-2 variants, a systematic analysis was conducted using serum samples collected up to 9 months after the third dose of vaccination from 432 healthcare workers [227]. To this end, different three-dose combinations of BNT162b2/Comirnaty (Pfizer-BioNTech), mRNA-1273/Spikevax (Moderna), and adenovirus-vector-based ChAdOx1-S/Vaxzevria (AstraZeneca-Oxford) were employed, with each eliciting an immune response against the S protein. Their findings suggest that while all these combinations elicit notable levels of nAbs against earlier SARS-CoV-2 variants, they are less proficient in generating nAbs against the newer BQ.1.1 and XBB.1.5 variants. This underscores the significance of continuously updating vaccines to address the emergence of new variants. Apart from that, these findings also emphasize the continuous necessity for genomic surveillance in order to track the appearance and dissemination of novel SARS-CoV-2 variants, and assess their impact on population immunity.

## 9. Key Challenges and Limitations of Antibody-Based Approaches

Amidst the global challenges posed by the SARS-CoV-2 virus, it is crucial to explore effective therapeutic solutions. Various therapies have received emergency authorization to combat COVID-19, aiming to prevent severe outcomes and transmission in high-risk individuals and COVID-19 patients. However, the effectiveness of certain antibody-based therapies, such as mAbs, faces limitations against emerging VOCs. Here, in the following sections, we discuss some key challenges and limitations of antibody-based approaches.

### 9.1. Risks of Antibody-Dependent Enhancement (ADE) for SARS-CoV-2 Antibodies and Their Implications

Antibodies that are unique to a particular virus are commonly recognized as antivirals and are involved in the prevention and management of viral infections in various contexts. The circumstances in which the presence of specific antibodies may be beneficial to the virus are referred to as the ADE of virus infection, hence promoting viral proliferation. ADEs pose a significant risk by intensifying the severity of infections, as evidenced across multiple viral illnesses like RSV, dengue, Zika, SARS, and MERS in humans [228,229,230]. Although ADE is observed in vitro for many viruses, there is limited evidence suggesting its development following SARS-CoV-2 infection, influencing the severity of COVID-19 [231,232]. ADE might be a factor, particularly in acute respiratory distress syndrome, which is a primary cause of fatality in severe COVID-19 cases. Some studies report a correlation between higher SARS-CoV-2 antibody titers in COVID-19 patients and increased disease severity [46]. Concerns also arise regarding pre-existing antibodies that act against other human coronaviruses, potentially leading to ADE by aiding cross-reactive recognition of SARS-CoV-2 in COVID-19 patients, even without viral neutralization [232,233]. Consequently, the potential for ADEs to result in SARS-CoV-2 infection raises considerable apprehension regarding the efficacy and safety of antibody-based vaccines and treatments [232,234]. In the context of viral infections, ADEs can be classified into two overarching categories: enhanced infection and enhanced immune activation. Greater infection rates are facilitated through the interaction between Fc and FcγR [232]. Extensive in vitro studies have focused on the mechanism through which ADE infiltrates phagocytic cells via FcγRIIA-mediated endocytosis. This process has been particularly explored concerning macrophage-tropic viruses like dengue virus and feline infectious peritonitis virus (FIPV) [235]. A study revealed that neutralizing mAbs MW01 and MW05, which are used to trigger ADE of SARS-CoV-2 pseudovirus in Raji and Daudi B cells, primarily relied on FcγRIIB rather than FcγRIIA. Remarkably, MW01 and MW05 exhibited significant adverse effects on Raji cells expressing only FcγRIIB. Furthermore, the ADE response was notably diminished or entirely eradicated in Daudi and K562 cells co-expressing both FcγRIIA and FcγRIIB [234]. Studies based on SARS-CoV have also documented that ADE can enter FcγRIIB-expressing B cells through endocytosis, which is facilitated by FcγRIIB [236,237]. These studies suggest that disrupting the Fc-FcγR interaction through an Fc mutation may serve as a potential strategy in order to counteract prospective ADE in vivo. While evidence shows that the loss of bivalent binding reduces ADE activity, a process facilitated by several SARS-CoV-2-neutralizing mAbs, the precise mechanism responsible for diminished monovalent ADE binding remains elusive. It might be possible that bivalent interactions can foster immune complex formation and increased cross-linking, which is possibly influenced by alterations in S-trimer conformation. However, further investigation is required to elucidate the relationship between mAb’s bivalent interaction and ADE [234]. Additionally, passive immunization or vaccination may trigger ADE through antibody transfer or production. Notably, passive immunization using hyperimmune plasma from COVID-19 convalescents has been found to be safe and effective, without inducing ADE [173]. While efforts are underway to mitigate the risk of ADE through the careful selection and design of therapeutic antibodies, thorough preclinical and clinical evaluation is imperative to ensuring their safety and efficacy.

### 9.2. Original Antigenic Sin Effect Restricts Vaccine Efficacy against SARS-CoV-2 VOCs

Upon encountering a foreign antigen such as a viral surface protein, the immune system initiates a primary immune response, generating specific antibodies and cytotoxic T lymphocytes. Some B and T lymphocytes become memory cells during this response, which is crucial for achieving future immunity. Upon subsequent exposure to the same antigen, memory cells trigger a faster and stronger immune response due to memory-cell activation. However, if the virus mutates, the original memory cells might not recognize it, leading to a primary immune response again. If the mutated antigen triggers the memory cells, it can overpower the response, though it cannot effectively neutralize the virus. This phenomenon, termed antigenic sin, or original antigenic sin (OAS), was first described in 1960 by Thomas Francis Jr. during influenza virus infections [238]. In the context of COVID-19 vaccines, particularly inactivated vaccines, antigenic sin could become a concern when individuals who have been vaccinated with a first-generation vaccine encounter SARS-CoV-2 VOCs. The immune system may predominantly respond to antigens present in the original vaccine, potentially reducing the effectiveness of the immune response against the VOCs [239]. In a study, Röltgen et al. investigated the antibody responses induced by various COVID-19 vaccines in comparison to those elicited by prior infection. It was observed that individuals infected with different viral variants produce antibodies which are specific to those variants, while those vaccinated with mRNA vaccines predominantly develop antibodies targeting the original Wuhan-Hu-1 [240]. A recent study found that thrice-vaccinated individuals showed weakened immune responses to mutated epitopes of the omicron variant of SARS-CoV-2. Pušnik et al. observed that, while T-cell responses to omicron’s mutated epitopes persisted due to vaccine-induced cross-reactivity, humoral and memory B-cell responses against the altered regions were weakened. This implies that prior vaccination could potentially predispose individuals to OAS if future variants evade vaccine-induced immunity [241]. This is indeed a consideration for the development of second-generation COVID-19 vaccines, where the vaccines are effective against emerging variants of SARS-CoV-2. Strategies such as incorporating multiple strains or antigens into the vaccine or designing vaccines that elicit broader immune responses may help to mitigate the risk of antigenic sin.

### 9.3. Long COVID or Post-COVID Conditions

A significant number of COVID-19 patients exhibit persistent symptoms beyond the acute phase of infection. Many individuals who have recovered from COVID-19 or have been vaccinated report experiencing long-term symptoms, also referred to as long-haul or long COVID infections. These symptoms can encompass deficits in the central nervous system, including brain fog, fatigue, and difficulty concentrating [242,243,244]. Researchers have not yet completely understood the causes of these chronic symptoms, but they may be related to changes in the immune system, chronic inflammation, or the persistence of virus particles in the body [38,245,246]. Understanding the Th1 immune response is vital in combating viral infections, including SARS-CoV-2. Th1 cells, a subset of CD4^+^ T cells, play a pivotal role in orchestrating immune defense against intracellular pathogens. They release cytokines such as IFN-γ, activating macrophages to eliminate intracellular pathogens. Moreover, cytotoxic CD8^+^ T cells identify and eliminate virus-infected cells, halting intracellular viral replication and preventing persistent infection [247]. Neutralizing antibodies, a crucial component of the humoral immune response, can block virions from infecting new cells. Although the exact function of nAbs in such instances remains unclear, preliminary research suggests that individuals with elevated levels of these antibodies may have a reduced risk of developing chronic illnesses [248,249]. A balanced Th1/Th2 response, fostering both cellular and humoral immunity, is crucial in effectively combatting SARS-CoV-2 [250,251].

Furthermore, a study led by Huang and Fishell reports that SARS-CoV-2 infection most frequently manifests itself as a condition of the central nervous system and can even persist for weeks or even months [252]. Later, Hirzel et al. found that COVID-19 patients experiencing neurological symptoms exhibited lower levels of nAbs compared to those without such symptoms. This observation suggests a potential role for nAbs in safeguarding against CNS complications in COVID-19 [253]. Therefore, nAbs are currently being studied to determine if there is a connection between long-haul COVID-19 symptoms and the potential for prevention or treatment using nAbs. In a study by Zou et al., neurological symptoms were found to be prevalent in 9.9% of recovered COVID-19 patients [254]. SARS-CoV-2-infected cells may cross the blood–brain barrier (BBB) and enter the brain through perivascular spaces, potentially triggering an immune response in patients with viral encephalitis [255]. Encephalopathy has been documented in 10.3% of the 901 COVID-19 patients studied, highlighting a wide array of neurological manifestations. The observed damage within the central nervous system (CNS) or peripheral nervous system (PNS) in these instances is thought to be caused directly by the virus itself or by the body’s innate and adaptive immune responses to SARS-CoV-2 infection [256]. Evidence suggests the existence of systemic inflammation in some COVID-19 patients, manifested by IgG synthesis within the intrathecal region and the presence of immunoglobulin proteins in cerebrospinal fluid (CSF) and serum [257]. Neuro-COVID patients exhibit depleted CD4^+^ T cells and dedifferentiated monocytes in their CSF, accompanied by increased interferon signatures that are indicative of a significant immune response, albeit less pronounced than that observed in virus-induced encephalitis [258]. Inflammatory cytokines, particularly those driven by type II interferon, may cause neuronal injury and result in long-term neurologic effects, including neurocognitive dysfunction [259]. CNS impairment might be associated with cytokine release syndrome (CRS), elevated serum IL-6 levels, and increased BBB permeability, as evidenced by hyperalbuminorrachia and heightened astroglial protein S100B [260]. The spread of hematogenous SARS-CoV-2 to the brain, facilitated by direct viral invasion and inflammatory reactions, could synergistically impact neurological function.

A plausible source of amyloid in COVID-19 infections is the combination of identified SARS-CoV-2 amyloid proteins and hypothesized potential amyloids [261,262]. Consequently, infection with SARS-CoV-2 might cause the host to generate both extracellular and intracellular amyloid deposits. Similar to the endogenous amyloids associated with dementia, the SARS-CoV-2 virion can infect various cell types in the brain, including neuronal and non-neuronal cells, suggesting a potential involvement of these deposits in dementia pathogenesis [263,264]. Certain forms of SARS-CoV-2 amyloid are hazardous and can directly alter brain function or exacerbate COVID-19-associated dementia [264]. Moreover, interactions between SARS-CoV-2 amyloid proteins and endogenous Aß and other amyloids may facilitate the deposition of additional amyloid types, potentially leading to dementia such as Alzheimer’s disease (AD) [265]. Considering their potential causative role in COVID-19-exacerbated dementia, SARS-CoV-2 amyloid proteins may represent disease-modifying targets [261,262,264]. Remarkably, these patients exhibit substantial variations in circulating myeloid and lymphocyte populations, including the prominent activation of peripheral B cells and a stronger humoral immune response against SARS-CoV-2. Elevated levels of non-classical monocytes and intermediate monocytes may disrupt the equilibrium of blood vessel substances, potentially leading to persistent inflammation, which is predominantly regulated by Th1 cytokines. The prevalence of decreased counts of CD4^+^ and CD8^+^ T cells, coupled with the diminished central memory of CD4^+^ T cells, indicates a distinctive immunological pattern, characterized by attenuated effector activity and aberrant immune responses [266].

## 10. Discussion and Future Perspectives

The global emergence of SARS-CoV-2 in late 2019 sparked a profound public health crisis, resulting in millions of deaths worldwide. Efforts to address the ongoing and potential future impacts of the COVID-19 pandemic have led to significant advancements in diagnostics, antibody-based treatments, and vaccines. These developments are crucial for effectively managing the spread of the virus and mitigating its impact on public health. Although qRT-PCR methods are considered the current gold standard, they come with several limitations. To address these constraints, diverse serological assays like ELISA, CLIA, and RDTs are being utilized to detect SARS-CoV-2-specific antibodies, particularly IgM and IgG [81,82]. The dynamic antibody levels in COVID-19 patients offer valuable clinical insights, especially for suspected cases with negative viral RNA tests or prior infection. However, cross-reactivity significantly affects the sensitivity and specificity of serological tests, posing a challenge to attempts at accurate diagnosis. Therefore, integrating serological tests with nucleotide-based qRT-PCR assays can markedly enhance the sensitivity and specificity of clinical diagnosis for COVID-19 and improve positive viral detection rates across diverse populations. However, to address the demands caused by any possible future pandemic, researchers are evaluating and developing new detection methods in order to continuously track biomarkers that are important for both individual and population health [267].

The ability of the humoral immune system to resist SARS-CoV-2 infection heavily relies on antibodies, which can also be transformed into potent treatments for COVID-19 [268]. nAbs and bAbs are linked to protection against symptomatic SARS-CoV-2 infection. As discussed previously, mAbs, convalescent plasma (CP), and hyperimmune intravenous immunoglobulins (IVIGs) are employed to treat COVID-19 patients, particularly those who are immunocompromised and prone to severe illness post SARS-CoV-2 infection [177]. However, the emergence of SARS-CoV-2 VOCs, including the highly transmissible sub-lineages of the Omicron variant, poses many challenges. These variants possess multiple mutations in the NTD and RBD of the spike protein, rendering them resistant to therapeutic mAbs and antibodies induced by mRNA-based SARS-CoV-2 vaccines or previous infections. These VOCs diminished or eliminated the ability of several antibodies, including those authorized for emergency therapeutic use, to effectively neutralize the virus [269,270,271,272,273]. Several reports indicate a significant decline in most of the mAbs’ efficacy against emerging Omicron subvariants [134,146,147,148,274,275]. Sotrovimab showcases the potential of antiviral mAbs in terms of granting long-term passive immunity. However, it susceptible to viral mutations. Hence, it is imperative to devise ways to develop antibodies that possess the ability to withstand viral evasion. The strategically development of different combinations of antibodies targeting different regions of the SARS-CoV-2 virus could potentially enhance protection against its variations [276,277]; however, such an approach increases manufacturing costs and demands higher dosing. To develop anti-SARS-CoV-2 spike antibodies with a broader spectrum of neutralization, alternative strategies have also been explored, such as the production of multispecific antibodies. To prevent immune escape via resistance mutations, various antibody-variable regions targeting distinct RBD sites can be combined to generate multispecific antibodies [278,279]. Such multispecific antibodies have demonstrated over 3000-fold greater efficacy on Omicron variants BA.1, BA.2, and BA.5 in terms of preventing virus escape compared to the most potent clinical antibody or parental antibody mixtures, as evidenced by several studies [280]. However, in the absence of humoral immunity, the inhibition of viral proliferation alone may not suffice for viral clearance. Therefore, combining antiviral compounds with anti-spike mAbs may also result in greater efficacy due to their distinct antiviral mechanisms [281].

In addition to treatments that target viral and host factors, a COVID-19 therapeutic regimen may also include drugs that specifically target viral enzymes. Remdesivir and ledipasvir are among the FDA-approved antivirals that target RNA-dependent RNA polymerase (RdRp) and they have been shown to have inhibitory effects against SARS-CoV-2 replication [282]. Furthermore, molnupiravir, which is also a viral RdRp inhibitor, exhibits promising efficacy in treating patients during the early stages of the disease. A recent phase 2a clinical trial, employing a double-blind, placebo-controlled randomized multicenter design, demonstrated the accelerated clearance of SARS-CoV-2 RNA and eradication of infectious virus in individuals with mild-to-moderate COVID-19 [283]. Notably, remdesivir, molnupiravir, and nirmatrelvir are among the antivirals approved for COVID-19 treatment, proving efficacy against some of the VOCs, including Omicron [284,285]. Paxlovid, a groundbreaking combination therapy comprising nirmatrelvir and ritonavir, has gained FDA approval as the first SARS-CoV-2 treatment targeting the main protease (M^pro^). A recent study by Papini et al. showcased Mpro61, a computationally designed M^pro^ inhibitor exhibiting synergistic effects with the SARS-CoV-2 antiviral molnupiravir in B6-K18-hACE2 mice. This promising finding positions Mpro61 as a viable alternative to Paxlovid, offering the potential to combat SARS-CoV-2 infections [286]. Combination therapies can also be utilized to treat COVID-19. There are various reasons for this, including synergy between compounds that target the same or different stages of the virus lifecycle or disease pathway, increased antiviral efficacy, and distinct mechanisms of action. A recent study demonstrated an excellent clinical response in immunocompromised patients with prolonged COVID-19 by combining two antivirals (mainly remdesivir and nirmatrelvir/ritonavir) with mAbs [287]. Thus, employing a blend of mAbs alongside concurrent small-molecule antiviral treatments, such as protease and replicase inhibitors, can mitigate the risk of resistance emergence, ensuring more robust and lasting efficacy against evolving viral strains [146,288]. However, the elevated risks associated with safety concerns, potential drug interactions, and adverse effects of these therapies may account for a portion of the limited number of subjects undergoing clinical trials for COVID-19 at this time. Thus, combination therapies represent a promising approach due to the propensity for drug resistance to individual compounds and the limited efficacy of current SARS-CoV-2 therapeutics.

In addition, our current understanding of COVID-19 is significantly limited by our incomplete grasp of its long-term health consequences. These include potential damage to vital organs, the immune system, mental health, and overall quality of life [289]. Conducting well-organized genome-wide expression analyses on extensive datasets, comprising both healthy individuals and those with pre-existing conditions, will be invaluable to efforts to gain deeper insights into the impact of COVID-19 infection and vaccination. Moreover, ensuring the availability of genome-wide study data on COVID-19 is essential for advancing the global understanding of the multifaceted aspects of SARS-CoV-2.

The approved vaccines also encountered various challenges due to the emergence of VOCs. This occurred owing to the short-lived nature of their protective antibodies and their limited cross-protection against mutated viruses [290]. To address these issues, strategies can be employed such as the administration of regular booster shots, using both homologous and heterologous vaccines to enhance the cross-reactive antibody levels and extend protection, which is similar to the annual influenza vaccination approach [291]. Re-administering immunizations is particularly crucial for vulnerable and marginalized groups, including elderly adults, organ transplant recipients, and cancer patients undergoing treatment for Delta and Omicron variants [292]. Efforts are underway to develop advanced vaccines against SARS-CoV-2, focusing on inducing immunity in the nasal and oral cavities, which are crucial entry points for the virus [293]. The IBIS, which is a live-attenuated mucosal pan-betacoronavirus vaccine, shows potential against SARS-CoV-1, SARS-CoV-2, and variants, boasting cross-protection and robust immune responses [294]. Additionally, efforts to enhance next-generation vaccine designs by incorporating additional epitopes from SARS-CoV-2 and other coronaviruses aim to broaden the spectrum of generated cross-protective antibodies, potentially offering increased protection against emerging variants a virus [295]. Given the susceptibility of the S protein to mutations, efforts are shifting towards more conserved regions like the N protein, which could potentially address concern about viral mutations and improve immune responses [296]. Overall, the development of next-generation vaccines for SARS-CoV-2 requires long-term immunity, which is capable of addressing viral mutations and promoting cross-protection [297]. The insights gleaned from these studies and approaches can further enhance the therapeutic potential of antibody-based treatments, providing valuable guidance for addressing future challenges posed by emerging mutant strains of SARS-CoV-2.

## Figures and Tables

**Table 1:** Table summarizing the available antibody-based serological tests used to detect SARS-CoV-2-specific proteins or antibodies.

Kit Name	Manufacturer	Test Type	Test Specimen	Target Antibody	Sensitivity	Specificity	Advantage	Limitations
EUROIMMUN Anti-SARS-CoV-2 ELISA (IgG)	Euroimmun	ELISA	Serum, plasma	IgG	91%	100%	Detects past exposure, potential immunity	May miss very early infections
WANTAI SARS-CoV-2 Ab ELISA	Beijing Wantai Biological Pharmacy	ELISA	Serum, plasma	IgM, IgG	96.7%	97.5%	Detects recent and current infections	May have lower sensitivity in earliest days of infection
Platelia SARS-CoV-2 Total Ab	Bio-Rad	ELISA	Serum, plasma	Total antibodies (IgG, IgM, IgA)	98%	~99%	Detects a broad range of antibodies for recent or past infection	May not differentiate between active and prior infection
MAGLUMI 2019-nCoV IgM/IgG	Snibe Diagnostics	CLIA	Serum, plasma	IgG, IgM	~90–95%	~98–99%	High sensitivity, automated, detects recent and current infection	Requires specialized lab equipment
LIAISON SARS-CoV-2 S1/S2 IgG	Diasorin	CLIA	Serum, plasma	IgG	>98%	>99%	Automated, highly sensitive, and specific	Requires specialized lab equipment
Atellica IM SARS-CoV-2 Total (COV2T)	Siemens Healthineers	CLIA	Serum, plasma	Total antibodies (IgG, IgM, IgA)	~95%	~99.5%	High-throughput, detects broader antibody responses	Requires specialized lab equipment
Abbott ARCHITECT SARS-CoV-2 IgG Immunoassay	Abbott	CLIA	Serum, plasma	IgG	>99%	>99%	Automated, high-throughput, excellent performance	Requires specialized lab equipment
Elecsys Anti-SARS-CoV-2	Roche Diagnostics	CLIA	Serum, plasma	Total antibodies	>99.5%	>99.8%	Detects past exposure or recent infection, excellent sensitivity and specificity	Requires specialized lab equipment
SGTi-flex COVID-19 IgG	Sugentech	CLIA	Serum, plasma	IgG	96.7%	100%	Quantitative results, automated and fast	May require regulatory approval, performance data are needed
QUANTA Flash SARS-CoV-2 IgG	Inova Diagnostics	CLIA	Serum, plasma	IgG	~98%	~99%	Detects past exposure or recent infection, good sensitivity and specificity	Requires specialized lab equipment
YHLO Biotech iFlash1800	Yhlo Biotech	CLIA	Serum, plasma	IgM, IgG	100% after 15 days post symptom	100%	Compact, versatile, can run various CLIA tests	Requires specific test kits for SARS-CoV-2
Panbio COVID-19 Ag Rapid Test Device	Abbott	LFIA	Nasal swab	Viral antigen	~95%	~99%	Rapid results, easy to use	Lower sensitivity than PCR in some cases
ACON Laboratories ACON SARS-CoV-2 IgG/IgM Rapid Test	Acon Laboratories	LFIA/rapid test	Fingerstick blood, serum, plasma	IgM, IgG	96.7% 98.8%	100% 97.5%	Rapid results, point-of-care	Lower sensitivity than lab-based tests, performance data are needed
LumiraDx SARS-CoV-2 Ab Test	Lumiradx	RDT	Nasopharyngeal specimen	IgG, IgM	>90%	97.8%	Rapid results, point-of-care	May require regulatory approval, performance varies by test version
BioPlex 2200 SARS-CoV-2 IgG	Bio-Rad	Multiplex immunoassay	serum, plasma	IgG (multiple targets)	>98%	>99%	Detects IgG on multiple SARS-CoV-2 proteins, excellent performance	Requires specialized lab equipment
CovAb™ SARS-CoV-2 Ab Test	Diabetomics	Presumed LFIA/rapid test	Saliva (oral fluid)	IgG, IgM, IgA	93.39–99.97%	>98%	Saliva-based, non-invasive	May require regulatory approval, performance data are needed
BD Veritor System for Rapid Detection of SARS-CoV-2	Becton, Dickinson And Company (BD)	RDT (Lateral Flow)/GICA	Nasal swab	Viral antigen	~84%	~99%	Fast results, portable system	Less sensitive than PCR in some cases

**Table 2:** List of monoclonal antibodies and interleukin inhibitors for the management of SARS-CoV-2.

Monoclonal Antibody	Developer	Authorization Status	Main Applications	Advantages	Disadvantages	Protein Targets
Casirivimab and Imdevimab (REGN-COV2)	Regeneron Pharmaceuticals	EUA 21 November 2021, for early therapy in outpatients at high risk of disease progression; restricted on 24 January 2022.	• Early treatment of mild-to-moderate COVID-19 in high-risk individuals to prevent hospitalization. • Post-exposure prevention (in limited high-risk scenarios).	• Reduced hospitalization rates—neutralizes SARS-CoV-2 virus effectively.	• Intravenous administration may require a healthcare facility. • Limited availability and distribution.	Bind to the receptor-binding domain (RBD)
Bamlanivimab and Etesevimab	Eli Lilly and Company	EUA 9 February 2021, for early therapy in outpatients at high risk of disease progression; restricted on 24 January 2022.	• Early treatment of mild-to-moderate COVID-19 in high-risk individuals to prevent hospitalization. • Post-exposure prevention (in limited high-risk scenarios).	• Effective against earlier SARS-CoV-2 variants. • Potential for both treatment and prevention in certain contexts.	• Loss of efficacy against newer variants like Omicron subvariants. • Intravenous administration required (less convenient than oral medications). • Potential for infusion-related or allergic reactions.	Spike protein (S1)
Sotrovimab	GlaxoSmithKline (GSK) and Vir Biotech	EUA 26 May 2021, for early therapy in outpatients at high risk of disease progression; withdrawn on 5 April 2022.	Treatment of mild-to-moderate COVID-19	• Demonstrated efficacy against certain SARS-CoV-2 variants. • Retains some activity against Omicron subvariants.	• Reduced effectiveness against certain SARS-CoV-2 variants • Limited availability and distribution	Spike protein (RBD)
Tixagevimab and Cilgavimab (AZD7442)	AstraZeneca	EUA 8 December 2021 for pre-exposure prophylaxis	Clinical trials for prevention and treatment	• Investigational product with potential for prevention and treatment.	• Single intramuscular injection may not be suitable for everyone. • Limited effectiveness against certain variants. • Risk of hypersensitivity reactions.	Spike protein (RBD)
Bebtelovimab (LY-CoV555)	Eli Lilly and Company	EUA 11 February 2022, for early therapy in outpatients at high risk of disease progression	Treatment of mild-to-moderate COVID-19	• Reduces the risk of hospitalization and death. • Retains activity against Omicron subvariants	• Reduced effectiveness against certain SARS-CoV-2 variants. • Limited availability and distribution. • Risk of hypersensitivity reactions.	Spike protein (S1)
Evusheld (AZD8895 and AZD1061)	AstraZeneca	Emergency Use Authorization (EUA)	• Pre-exposure prophylaxis in certain individuals	• Offers protection against SARS-CoV-2 for several months	• Limited availability and distribution	Spike protein (RBD)
Tocilizumab	Developed for other autoimmune conditions	Variable based on local guidelines	• Can improve the patient’s condition from cytokine storm syndrome by inhibiting the IL-6 receptors.	• Targets the inflammatory response in severe cases	• Potential risk of infections, including reactivation of latent tuberculosis—long-term safety concerns need further investigation.	IL-6
Ronapreve (Casirivimab and Imdevimab)	Roche and Regeneron	• Approved in the European Union, Japan, New Zealand, and Switzerland. • Conditionally approved in Australia and the United Kingdom, • Emergency or temporary pandemic use in such as Canada and the US.	• Treatment of COVID-19 in certain populations	• Diminished potency versus the SARS-CoV-2 VOC, Omicron (B.1.1.529) while it has shown to retain its activity against all other main variants of concern, including Delta (B.1.617.2).	• Intravenous administration may require a healthcare facility. • Limited availability and distribution. • Infusion-related reactions and injection site reactions.	Spike protein (RBD)
BRII-196 and BRII-198	Brii Biosciences	Investigational	• Can rapidly increase anti-SARS-CoV-2 IgG 32 times	• Improved clinical status in patients with SARS-CoV-2 delta variant infection	• Investigational status, not yet authorized for widespread use—limited data on efficacy and safety	Spike protein (S1 and RBD)

**Table 3:** Table representing SARS-CoV-2 variants of concern (VOCs) along with all the associated information regarding the originating country, genetic mutations, transmission rate, and vaccine efficacies (%).

SARS-CoV-2 Variants of Concern (VOCs)	Country of Origin	Mutations (w.r.t. Wild Type SARS-CoV-2)	Transmissibility	Vaccine Efficacy (%)					
Vaccines				BNT162b2	mRNA-1273	Covaxin	ZyCov-D	AZD1222, AstraZeneca	Ad26.CoV2.S
Vaccine Platform Used				mRNA	mRNA	Inactivated	DNA	Viral Vector	Viral Vector
Alpha (B.1.1.7)	United Kingdom	N501Y, A570D, P681H, T716I, S982A, D1118H	30–50%	78–95%	84–99%	~70%	~66%	~90%	~86%
Beta (B.1.351)	South Africa	K417N, E484K, N501Y, Y453F, D614G	50%	~75%	~96%	-	-	~10%	60%
Gamma (B.1.1.28.1)	Brazil	L452R, P323L, T190S, K417N, E484K	30–40%	-	79%	-	-	-	~68%
Delta (B.1.617.2)	India	L452R, P681R, T716I, A222V, G142D, R191K, K417N, E484K, N501Y	80–90% more contagious than the alpha variant.	45–79%	76–84%	~65%	-	60.75%	-
Omicron (B.1.1.529)	Botswana and South Africa	N501Y, S477N, T478K, G446S, K417N, E484K, N679K, P681R, R203K, A222V, D614G, H655Y, N856K, P1057S, L452R, F486V, Q493K, G496S, S498R, Y505H, T547K, D614G, B1176T, 69–70del, 144–145del, 211–214del	The most transmissible VOCs	~65%	~71%	-	-	~62%	~80%

## Data Availability

The study did not report any new results or data.
